# Developing mRNA Nanomedicines with Advanced Targeting Functions

**DOI:** 10.1007/s40820-025-01665-9

**Published:** 2025-02-21

**Authors:** Ji Wang, Lijun Cai, Ning Li, Zhiqiang Luo, Haozhen Ren, Bing Zhang, Yuanjin Zhao

**Affiliations:** 1 Department of Radiology, Nanjing Drum Tower Hospital, Medical School, Nanjing University, Nanjing, 210008 People’s Republic of China; 2 State Key Laboratory of Bioelectronics, School of Biological Science and Medical Engineering, Southeast University, Nanjing, 210096 People’s Republic of China; 3 Department of Hepatobiliary Surgery, Hepatobiliary Institute, Nanjing Drum Tower Hospital, Medical School, Nanjing University, Nanjing, 210008 People’s Republic of China

**Keywords:** mRNA, Delivery system, Targeted therapy, Diseases, Precision medicine

## Abstract

The emerging messenger RNA (mRNA) nanomedicines have sprung up for disease treatment. Developing targeted mRNA nanomedicines has become a thrilling research hotspot in recent years, as they can be precisely delivered to specific organs or tissues to enhance efficiency and avoid side effects. Herein, we give a comprehensive review on the latest research progress of mRNA nanomedicines with targeting functions. mRNA and its carriers are first described in detail. Then, mechanisms of passive targeting, endogenous targeting, and active targeting are outlined, with a focus on various biological barriers that mRNA may encounter during in vivo delivery. Next, emphasis is placed on summarizing mRNA-based organ-targeting strategies. Lastly, the advantages and challenges of mRNA nanomedicines in clinical translation are mentioned. This review is expected to inspire researchers in this field and drive further development of mRNA targeting technology.

## Highlights

- The structure and modification strategies of messenger RNA (mRNA), along with advanced delivery carriers, are thoroughly reviewed to showcase their role in targeted drug delivery.
- Recent advancements in mRNA nanomedicines, focusing on targeted strategies for various organs, are summarized to illustrate their therapeutic potential.
- The major challenges and future perspectives for the clinical translation of targeted mRNA nanomedicines are discussed, emphasizing solutions to biological barriers.

## Graphical abstract

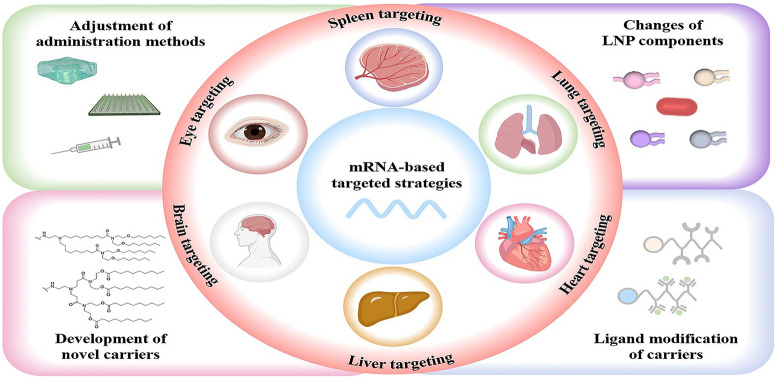

## Introduction

Messenger RNA (mRNA), which emerges as a beacon in the field of nanomedicine, has sparked widespread interest in both academic and commercial circles [1–5]. mRNA nanomedicines can guide cell factories to translate customized mRNA into functional proteins and generate specific immunological reactions for disease treatment [6–8]. Notably, the remarkable flexibility of mRNA synthesis allows for adjustments and customization to suit the therapeutic requirements of different diseases, making them powerful candidates for disease treatment [9]. Compared with traditional gene therapy, mRNA-based therapy circumvents the potential risks associated with genome insertion, which ensures the safety of treatment [10]. Also, mRNA nanomedicines can achieve protein synthesis in the cytoplasm, thus avoiding the drug metabolism issues faced by traditional chemical medicines [11]. Furthermore, the short production cycles and low costs of mRNA nanomedicines facilitate extensive manufacturing and global distribution, particularly beneficial for regions with limited medical resources [12]. Based on these features, mRNA-based therapy has garnered considerable attention and investment, heralding the arrival of a new medical era.

Despite a series of achievements, the efficiency of mRNA nanomedicines remains unsatisfactory because they encounter barriers including circulation barriers, endothelial barriers, and intracellular barriers. These challenges lead to accumulation in the liver after local or systemic administration, resulting in inevitable drug loss and reduced efficacy [13–16]. In view of this, scientific attention is concentrated on developing targeting technologies for delivering mRNA nanomedicines to specific sites [17–19]. One common targeting strategy involves adjusting the selection and proportion of lipid nanoparticles (LNPs) to enhance delivery efficiency and organ selectivity [20–22]. Another strategy is to develop new carriers with special chemical structures that can form stable nanoparticles under appropriate conditions, which increases the uptake level of targeted cells [23–27]. Furthermore, targeting delivery can also be achieved by modifying specific biological molecules or ligands on the surface of delivery vehicles [28, 29]. In addition to these approaches, many other targeting strategies are continuously being developed, all of which are expected to improve the delivery accuracy and therapeutic efficacy of mRNA nanomedicines, thereby enhancing their potential for clinical application.

**Fig. 1:**
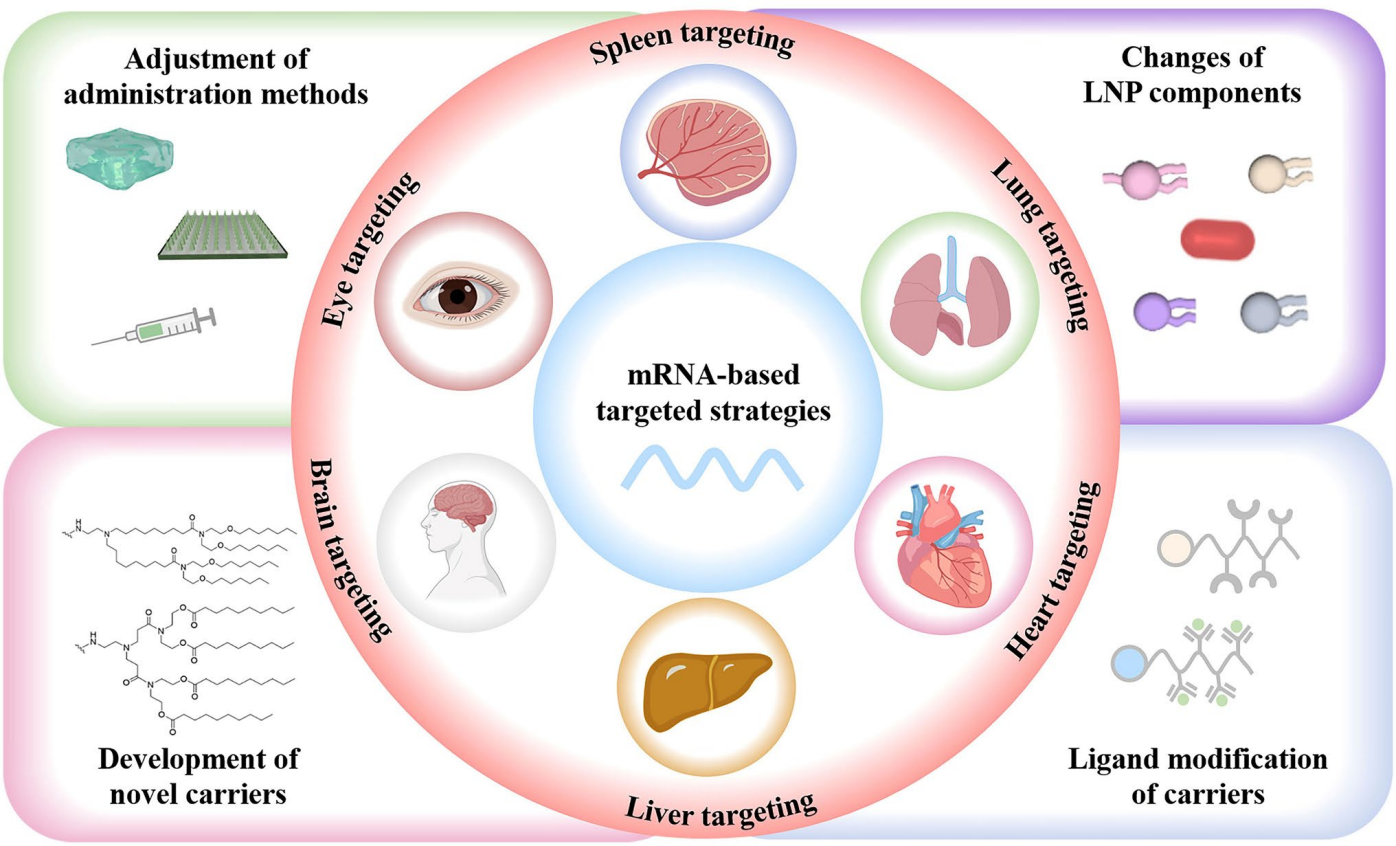
Advanced strategies for achieving precise mRNA delivery. To achieve organ targeting, strategies like adjusting administration methods, changing LNP components, developing novel carriers, and functionalizing carriers with ligands can be used. These methods allow mRNA to specifically target various organs

Considering the swift advancement of mRNA nanomedicines and their targeting technologies, this review seeks to offer a thorough overview with a specific focus on the latest research progress. We begin with clarifying the composition and working principles of mRNA nanomedicines and highlighting the delivery carriers associated with targeted therapy. Following this, we provide a basic summary of the mechanisms of three different targeting approaches (passive targeting, endogenous targeting, and active targeting) and also describe the biological barriers that mRNA nanomedicines face within the body. Particularly, we summarize recent targeting strategies for specific organs or sites, such as lung targeting, spleen targeting, heart targeting, brain targeting, and tumor targeting (Fig. 1). Lastly, we address the challenges that mRNA nanomedicines face as they transition from laboratory research to clinical practice and present insights into their future applications.

## mRNA Nanomedicines

### Structure and Modification of mRNA

**Fig. 2:**
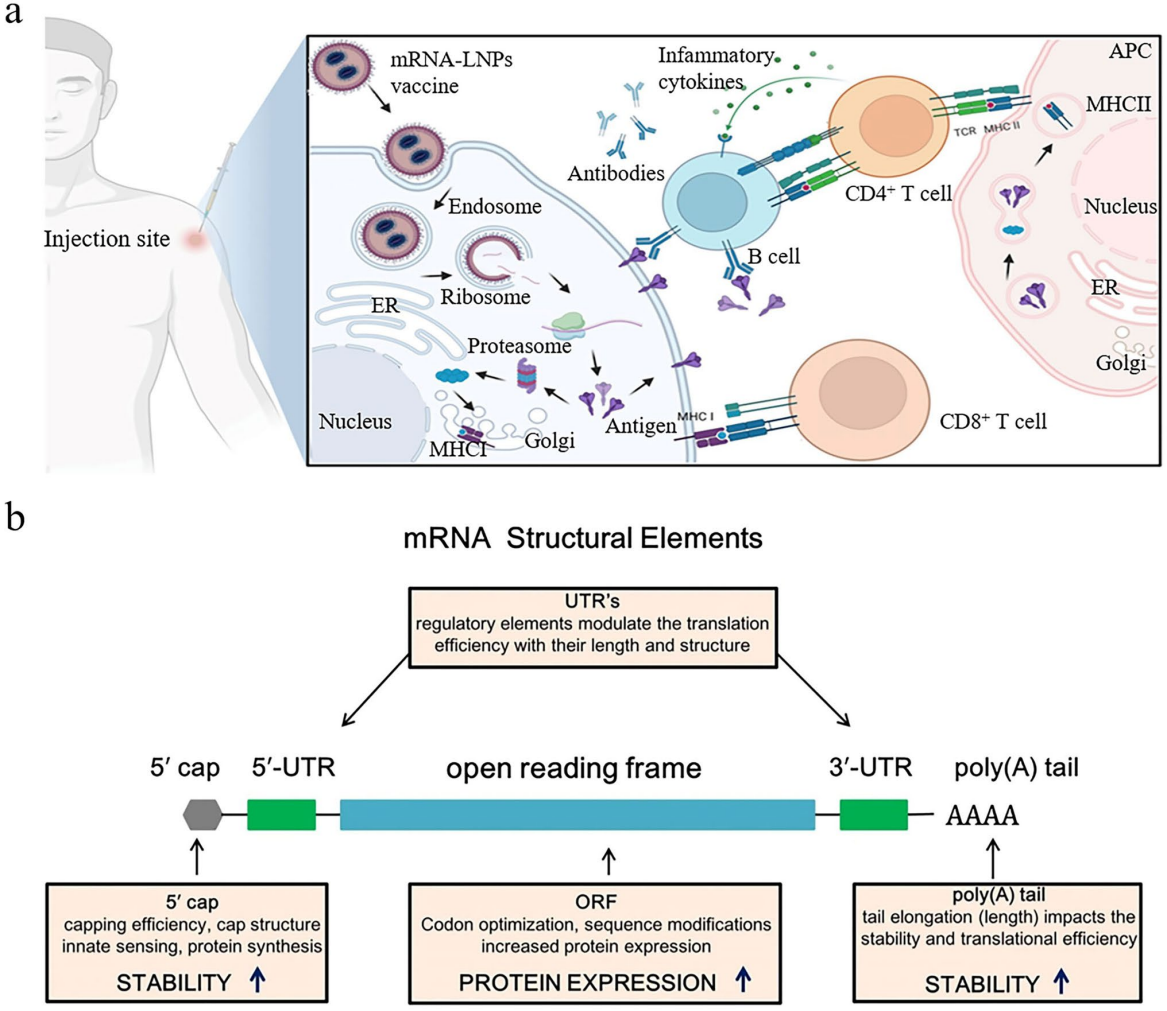
Introduction to the mechanism of mRNA vaccines and the structure of mRNA. **a** As a typical representative of mRNA therapeutics, mRNA vaccines exert their effects by injecting vector–mRNA complexes encoding pathogen antigen proteins into the human body. These vector–mRNA complexes utilize the host cells' translational machinery to synthesize the corresponding antigen proteins. After production, these proteins are displayed on the surface of host cells, where they are detected by the immune system. This behavior activates the immune response, leading to the generation of specific antibodies and other defense mechanisms. Reproduced with permission from Ref. [33]. Copyright 2023, MDPI. **b** Structure of mRNA is primarily composed of five elements, which work together to ensure the stability, translation efficiency, and functionality of the mRNA. Reproduced with permission from Ref. [42]. Copyright 2021, Springer Nature

mRNA nanomedicines, with their flexible design and extensive application potential, have demonstrated significant therapeutic prospects in modern medicine [30–32]. A successful example is the mRNA vaccines used in the field of infectious diseases. mRNA vaccines, as an innovative vaccine technology, introduce synthetic mRNA to instruct human cells to synthesize specific viral proteins and further elicit dual immune responses (Fig. 2a) [33]. Notably, these vaccines do not contain live viral components, which makes them highly safe [34]. During the Coronavirus Disease 2019 (COVID-19) pandemic, mRNA vaccines such as Pfizer–BioNTech's BNT162b2 and Moderna's mRNA-1273 show strong effectiveness in preventing infection and reducing the number of severe cases, becoming essential tools to fight against the pandemic [35–38]. However, the success of this technology is not coincidental but rather the result of over 60 years of exploration and development.

Endogenous mRNA, first discovered by Brenner and colleagues in 1961, is a kind of biomolecule with a simple structure but highly specialized functions [39]. The sequence and length of mRNA vary depending on the protein it encodes, whereas its basic structural features are largely similar across all eukaryotic organisms. As shown in Fig. 2b, the fundamental structure of mRNA includes the following parts: a cap structure, untranslated regions, an open reading frame (ORF), and a poly(A) tail [40–42]. These components have diverse functions and finely regulate the process of gene translation through complex interactions, thereby synergistically promoting efficient mRNA expression. The cap structure is located at the 5' end of mRNA and can be added during or after transcription. It protects the mRNA from nuclease degradation and signals ribosome recognition, ensuring protein synthesis initiation [43]. The non-coding regions of mRNA include the 5' untranslated region (5' UTR) and the 3' untranslated region (3' UTR). These regions do not have the capacity to encode proteins but are crucial in the regulation of gene expression. Located between the 5' cap and the coding region of the mRNA, the 5' UTR regulates translation efficiency and ribosome binding [44]. Meanwhile, the 3' UTR, found between the protein-coding region and the polyadenylation tail, contributes to localization, translation control, and interactions with microRNAs [45]. These untranslated regions ensure that genes are expressed at the appropriate levels, times, and locations through various mechanisms. The ORF is located between the 5' UTR and the 3' UTR, and it contains the genetic information necessary for synthesizing specific proteins [46]. By reading this sequence, ribosomes can translate it into the corresponding amino acid chain, further forming functional proteins. Reportedly, the introduction of chemically modified nucleotides or codon optimization in the ORF is beneficial for enhancing mRNA expression levels [47]. At the 3' end, mRNA typically features a poly(A) tail composed of dozens to hundreds of adenosine residues. This poly(A) tail serves to enhance mRNA stability, prevent degradation, and regulate translation efficiency, whether added post-transcriptionally or included in sequence design [48, 49].

**Fig. 3:**
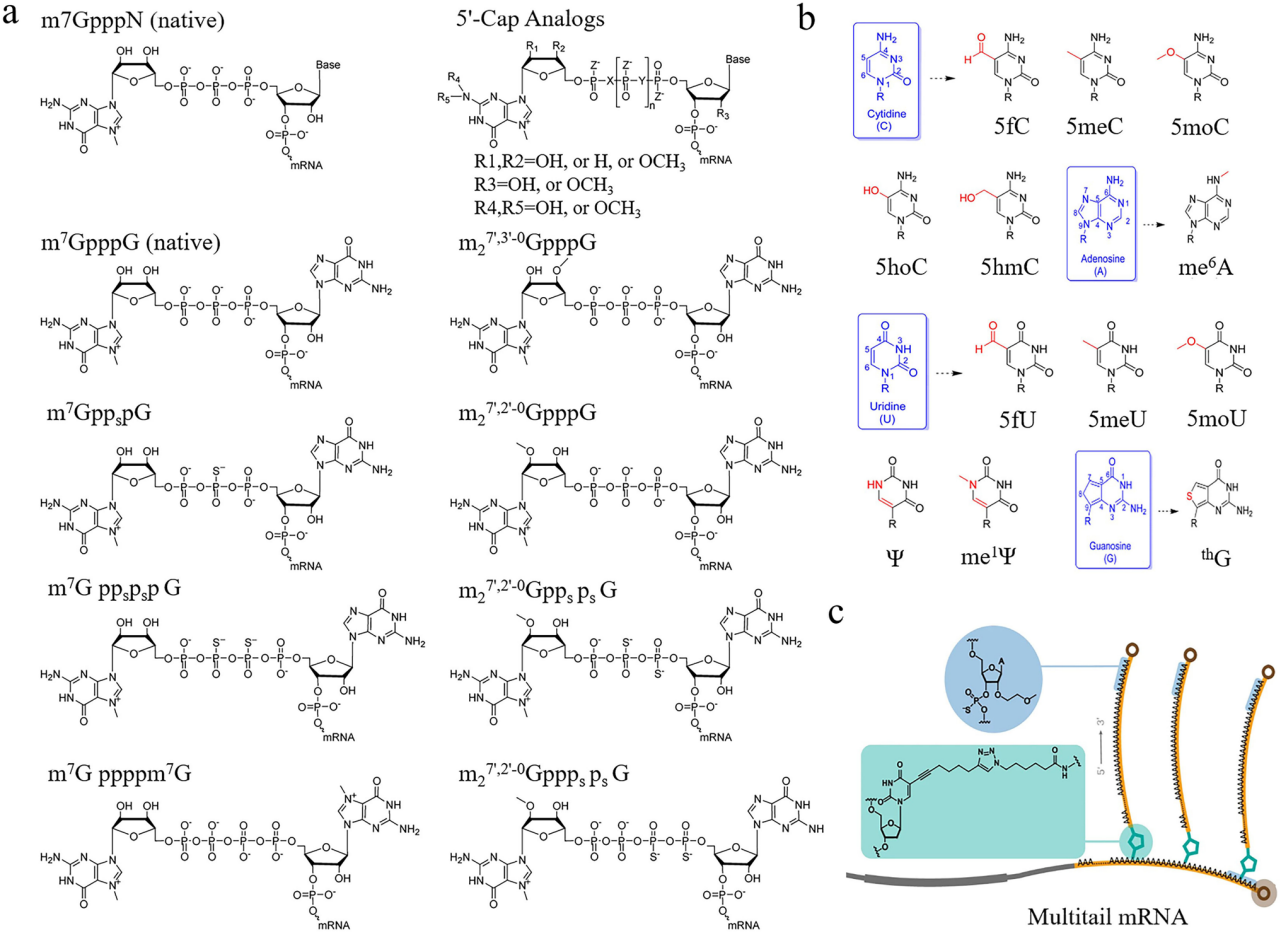
Modulation strategies for mRNA structure. **a** Chemical structures of 5' cap analogs. Reproduced with permission from Ref. [56]. Copyright 2020, Elsevier. **b** Chemically modified nucleotides are used to improve mRNA properties, including stability, translation efficiency, and immunogenicity. **c** mRNA molecules with branched poly(A) tails demonstrated longer expression durations compared to conventional mRNA, enhancing their potential in various applications. Reproduced with permission from Ref. [74]. Copyright 2024, Springer Nature

The mRNA modification strategy involves multiple critical regions to ensure its stability, translation efficiency, correct cellular localization, etc. In the 5' cap structure of mRNA, there are different forms of modifications, primarily including cap 0, cap 1, and cap 2 structures [50–52]. The cap 0 structure is the most basic form, where 7-methylguanosine is linked to the 5' end of the first nucleotide via a 5'-5' triphosphate bridge. The cap 1 structure builds upon cap 0 by further adding a methyl group at the 2'-hydroxyl position of the first nucleotide. The cap 2 structure extends cap 1 by also methylating the 2'-hydroxyl position of the second nucleotide as well. These progressive modifications not only enhance mRNA stability and translation efficiency but also are critical for immune recognition. Additionally, some 5' caps, such as S analogs, 2S analogs, and anti-reverse cap analogs (ARCA), have shown promising performance in preclinical trials (Fig. 3a) [53–56]. According to Strenkowska et al., a novel 2S analog was developed for cap modification, and it was demonstrated to exhibit a high affinity for the protein eukaryotic initiation factor 4E, which is associated with mRNA stability [57].

For the optimization of non-coding regions, the following guidelines can be considered: (1) Avoid excessively long non-coding sequences [58]. An overly long 5′ UTR may hinder the binding of the ribosome to the 5′ cap, affecting the translation progress of mRNA. Meanwhile, an extended 3′ UTR can destabilize transcripts by heightening the presence of microRNA binding sites, which leads to an increased risk of mRNA degradation. (2) The Kozak sequence in the 5′ UTR is crucial for start codon recognition [59]. (3) Incorporating some validated protein sequences into the non-coding regions, such as α-globin and β-globin, can extend the half-life of mRNA and increase protein expression levels [60]. (4) Reduce the frequency of AU-rich elements appearing in the 3' UTR, as these elements mediate mRNA decay [61]. Recently, with the continuous development of artificial intelligence, utilizing machine algorithms to design and optimize UTRs has also become a research hotspot. For instance, a pivotal study employed the Optimus 5-Prime algorithm platform for the de novo design of 5' UTRs [62]. The evidence suggested that mRNAs with algorithm-designed 5' UTRs improved gene editing efficiency across multiple dosage groups.

Nucleotide modification is a highly promising strategy to enhance ORF performance [63–65]. These modifications significantly impact mRNA fate and optimize gene expression and regulatory mechanisms. Conversely, unmodified mRNA faces issues with stability, translation efficiency, transport, localization, and immune responses. As early as 2008, Karikó and Weissman reported that incorporating modified nucleotides into transcripts deactivated most Toll-like receptors (TLR) [66]. Specifically, the incorporation of pseudouridine not only inhibited RNA-mediated immune activation but also enhanced mRNA expression levels. It must be mentioned that this pseudouridine substitution strategy has been used in two Food and Drug Administration (FDA)-approved mRNA vaccines [67, 68]. Given the advantages of modified nucleotides, new types (e.g., 5fC, 5hmC, 5fU) have been developed, as shown in Fig. 3b**.** In the context of molecular biology, optimizing codons is also a crucial method for enhancing ORF performance [69–71]. Codon optimization typically involves following specific rules, such as choosing codons that are most suitable for the host organism based on their frequency and efficiency during translation [72]. Avoiding rare codons can help reduce translation delays and errors. Additionally, it is essential to balance the optimized protein’s structural stability and functional activity to ensure proper folding and expression in biological systems. In the development of COVID-19 mRNA vaccines, a study used the algorithmic system LinearDesign to screen and optimize the mRNA sequences. Compared to non-optimized mRNA vaccines, the optimized mRNA vaccines demonstrated superior stability, higher protein expression levels, enhanced humoral immune response, and improved cellular immunity [1].

Designing the length and structure of the poly(A) tail can also effectively improve the expression level, duration, and stability of mRNA. For example, mRNA with a poly(A) tail of 120 adenosines exhibited higher expression levels compared to tails of 16, 42, and 51 adenosines (Fig. 3c) [73]. A recent research developed mRNA with multiple branched tails that produced luminescence signals 4.7 to 19.5 times higher than conventional mRNA and maintained expression for 14 days [74]. Reportedly, inserting a short linker or cytidine sequence between consecutive adenines also contributes to bolstering the functionality of mRNA within cells [75, 76]. Despite these optimization strategies appearing promising, their applicability and safety across different cell types and biological systems still require further validation. In conclusion, the modification of mRNA structure has brought significant innovations and advancements in gene regulation, providing strong support for the development of efficient gene therapies.

### mRNA Carriers Involved in Targeted Delivery

mRNA, as a highly fragile and negatively charged molecule, has difficulty in entering cells directly. It requires a delivery system to effectively transport it into cells and further perform biological functions. However, designing, preparing, and optimizing delivery systems are a complex task that requires a thorough understanding of chemistry, materials science, cell biology, and nanotechnology. Specifically, conventional carriers typically only passively deliver mRNA to the liver, making it challenging to target other desired organs or tissues. To overcome these issues, researchers are exploring novel delivery systems and related targeting technologies, such as lipid-based carriers, polymer-based nanocarriers, exosome carriers, and biomimetic carriers. These developed carriers not only improve mRNA stability and delivery efficiency but also facilitate organ-specific targeting. Therefore, this section focuses on vectors related to targeted mRNA delivery, providing a detailed discussion about their design, preparation, and potential in clinical applications.

#### Lipid-Based Carriers

**Fig. 4:**
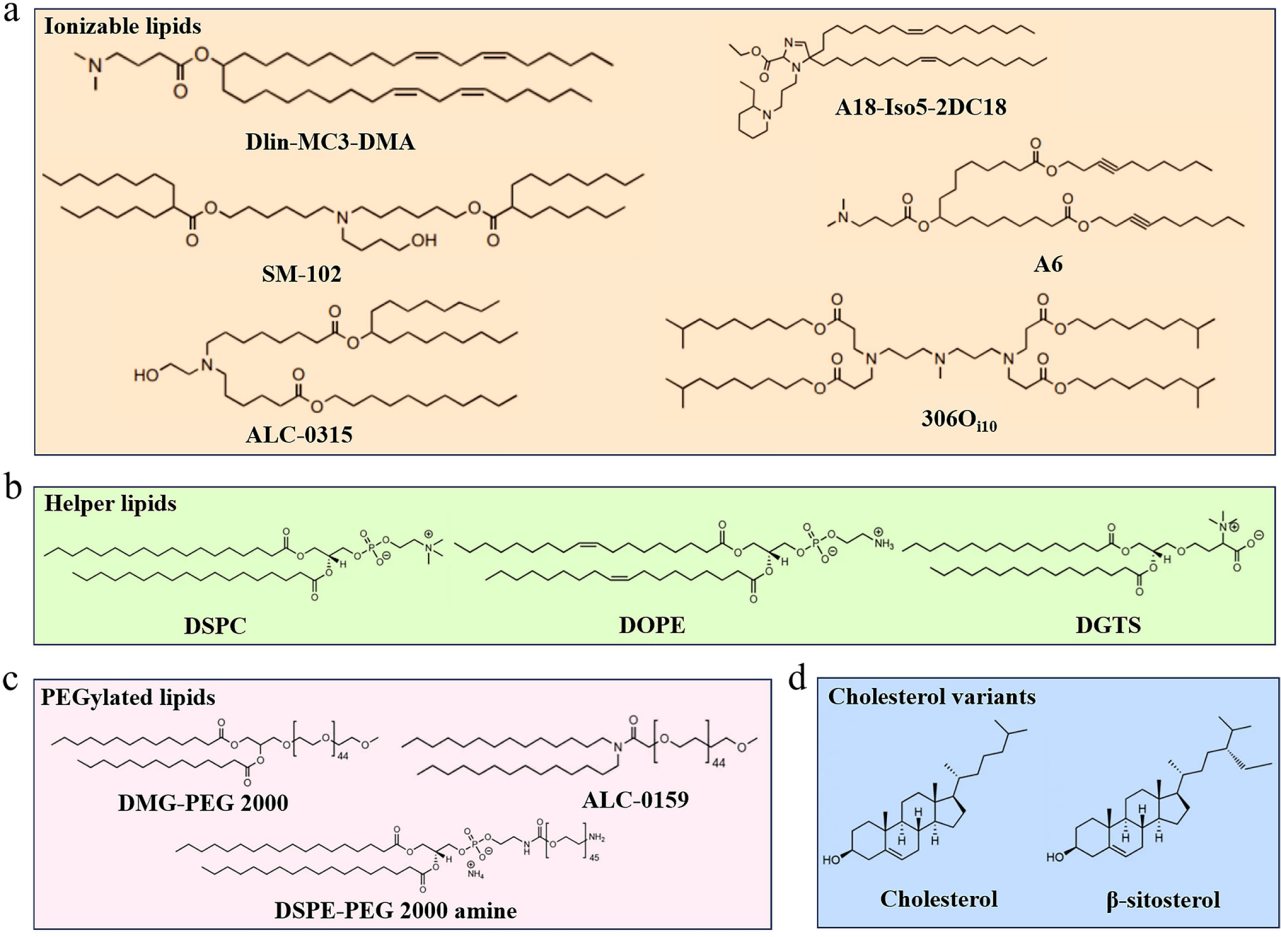
Components of LNP delivery systems are as follows: **a** ionizable lipids, **b** helper lipids, **c** PEGylated lipids, and **d** cholesterol. Each category includes some representative lipids

As efficient drug delivery systems, lipid-based nanocarriers have been researched for over 60 years. Starting with liposomes, first discovered by Bangham, a series of lipid-based delivery systems have been developed, including LNPs, lipid–polymer hybrid nanoparticles (LPNs), solid lipid nanoparticles (SLNs), lipid-like nanoparticles (LLPs), nanostructured lipid carriers (NLCs), and lipoprotein particles (LPTs), etc. [77–80]. Among these, LNP carriers have been particularly prominent in combating the COVID-19 pandemic, as the FDA-approved mRNA vaccines rely on LNPs as carriers [81]. LNPs are nanoscale carrier systems composed of different lipid molecules, including ionizable lipids, polyethylene glycol (PEG) lipids, helper lipids, and cholesterol [82], as shown in Fig. 4a–d. These lipid molecules work synergistically to form a protective spherical nanostructure that shields mRNA from degradation while facilitating its effective penetration through the cell membranes and subsequent intracellular release. Reportedly, ionizable lipids are crucial for the encapsulation and release of mRNA, as they change charge in response to pH variations, aiding endosomal escape [83]. Cholesterol is included to stabilize the LNP structure and enhance its fluidity and integrity. Furthermore, incorporating helper lipids helps to form a lipid layer that mimics the natural cell membrane and promotes cellular uptake. PEGylated lipids extend the circulation time of LNPs by reducing opsonization and clearance of the immune system. Given these features, it can be seen that the design of LNPs is highly flexible, which is unmatched by other traditional carriers. Typically, LNP-based mRNA nanomedicines are readily absorbed by the liver. By contrast, adjusting the composition and structure of LNPs allows for the optimization of biological distribution, stability, and intracellular delivery efficiency. This adjustment includes optimizing the properties of ionizable lipids, introducing specific surface-modified PEG lipids, and changing the type of phospholipids, among others. Ultimately, the ability to fine-tune the components of LNPs allows for the development of highly specialized and effective mRNA delivery systems tailored for specific therapeutic needs.

**Fig. 5:**
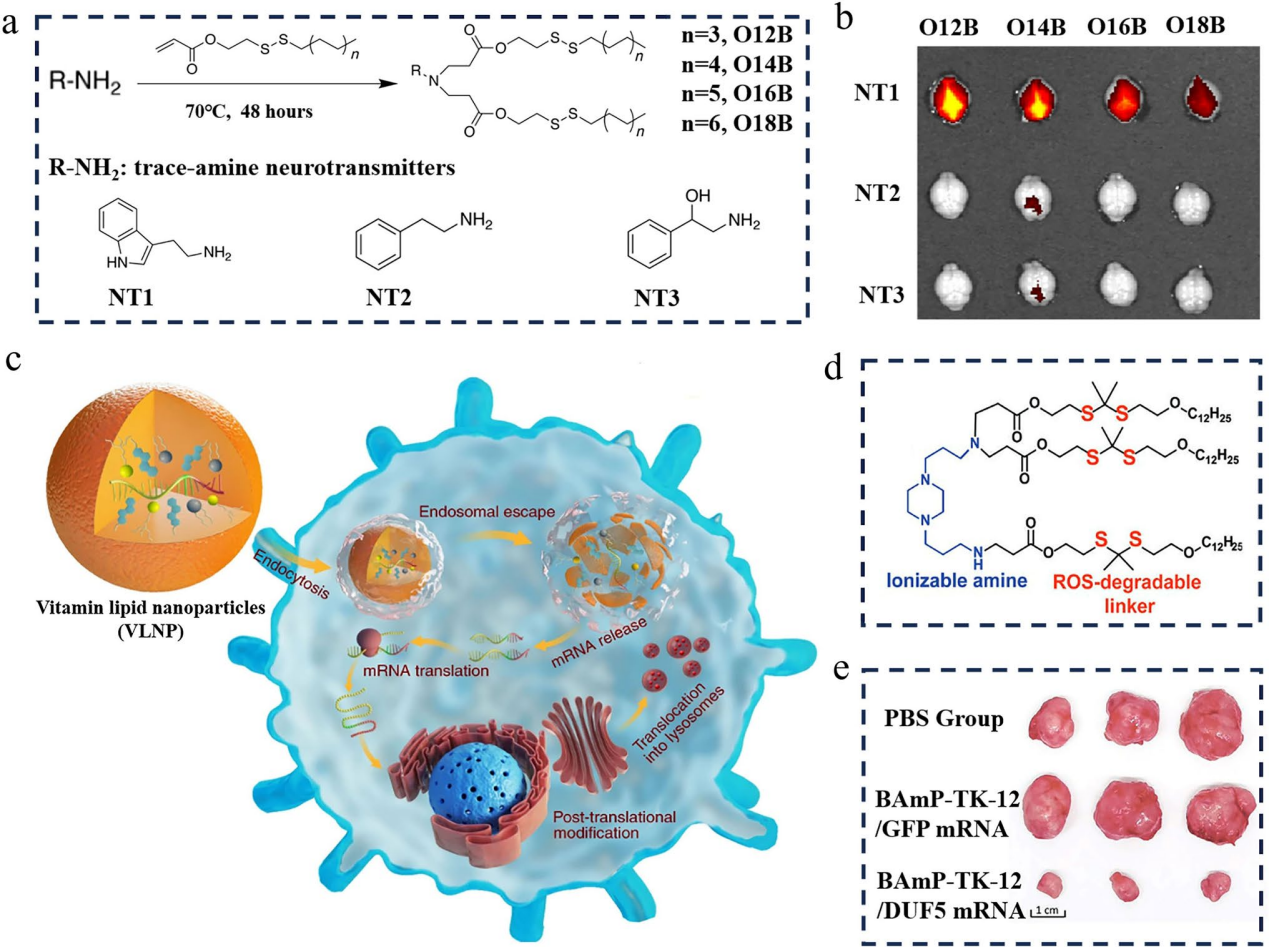
Introduction to ionizable lipids with targeting functions. **a** Structure of neurotransmitter-derived ionizable lipids. **b** Using neurotransmitter-derived LNP delivery systems, mRNA nanomedicines can penetrate the BBB as a large amount of biological signaling appeared in the mouse brain following intravenous administration. (**a, b**) Reproduced with permission from Ref. [87]. Copyright 2020, American Association for the Advancement of Science. **c** LNP formulations containing vitamin-modified ionizable lipids are designed to target macrophages for combating sepsis. Reproduced with permission from Ref. [89]. Copyright 2020, Springer Nature. **d** Structure of ROS-responsive ionizable lipids. **e** After treatment with ROS-responsive LNP nanomedicines, colorectal tumors in mice were significantly inhibited compared to other experimental groups. (**d, e**) Reproduced with permission from Ref. [90]. Copyright 2022, Wiley − VCH

Ionizable lipids are the most critical lipids in the LNP delivery systems, usually accounting for up to 50% [84]. As an upgraded version of traditional permanent cationic lipids such as 1,2-dioleoyl-3-trimethylammonium-propane (DOTAP), ionizable lipids maintain high delivery efficiency while exhibiting significantly reduced cytotoxicity. These lipids have a near-neutral charge in neutral or mildly alkaline environments but become protonated and carry a positive charge in acidic environments. This property allows them to effectively form complexes with negatively charged nucleic acid molecules, shielding them against breakdown and promoting cellular entry. Upon entry into the cell's acidic endosomal environment, these lipids can protonate again, facilitate endosomal rupture, and release the nucleic acids for effective gene expression or protein production. It is worth mentioning that DLin-MC3-DMA, SM-102, and ALC-0315, as three FDA-approved ionizable lipids, have similar overall chemical structures, mainly consisting of an amino head, linker, and hydrophobic branched tail [85, 86]. From a perspective of chemical structure, exploring the structure–activity relationship of ionizable lipids can help mRNA nanomedicines avoid accumulation in the liver and better target to specific organs. Inspired by neurotransmitter-like drugs, a study designed a series of neurotransmitter-derived ionizable lipids through the Michael addition reaction (Fig. 5a) [87]. After mixing with other lipids, the resulting neurotransmitter-derived LNPs can effectively carry cargo (nucleic acids, amphotericin B, or protein drugs) across the blood–brain barrier (BBB) without additional targeting ligands (Fig. 5b). At present, delivering biologics to primary T lymphocytes poses significant technical challenges. To address this issue, Zhao and his colleagues synthesized a library of imidazole-based lipids for targeting CD8^+^ T cells [88]. Furthermore, they optimized the length and chemical structure of the lipid tails, finding that lipid tails containing long carbon chains and disulfide bonds had higher affinity for T cells. Considering that functionalized macrophages can inhibit sepsis, Hou et al. developed vitamin-grafted ionizable lipids with the aim of delivering therapeutic mRNA to macrophages (Fig. 5c) [89]. It was observed that vitamin C-modified LNPs exhibited the highest delivery efficiency in RAW264.7 macrophage cells compared to LNPs modified with other vitamins, demonstrating ideal therapeutic effects in sepsis treatment. Additionally, by introducing reactive oxygen species (ROS)-responsive thioketal units into the tails of ionizable lipids, mRNA can be selectively transported to tumor cells and inhibit the growth of colorectal tumors (Fig. 5d, e) [90]. According to this report, it is the first instance of designing ROS-responsive LNPs, offering more candidate options for cancer therapy. Therefore, the above studies indicate that an in-depth investigation of the structure–activity relationship of ionizable lipids is beneficial for improving the targeting performance and delivery efficiency of mRNA nanomedicines.

Although PEG lipids constitute only 1% to 2% of the total lipids in LNPs, their contribution to the functionality and stability of the nanoparticles is substantial [91]. By forming a protective hydration shell, PEG lipids can prevent nanoparticles from being rapidly cleared and extend their circulation time in the blood. Secondly, the PEG chains provide electrostatic and steric hindrance effects, which reduce the aggregation and fusion of nanoparticles. Most importantly, targeted delivery to specific cells or tissues is possible by modifying the PEG lipids with specific targeting ligands. For example, by attaching small molecules, peptides, or antibodies to the PEG chains, the engineered LNPs can more effectively deliver mRNA to specific cell types, such as T cells, photoreceptor cells, and bone marrow cells. In the design of targeted PEG lipids, the maleimide (Mal) reaction is crucial as it allows Mal groups in PEG-derived lipids to specifically react with thiol groups on targeting ligands. The advantages of this reaction lie in its high selectivity and efficiency, which facilitates stable attachment of target molecules without disrupting the PEG chain structure. Recently, a study from the Mitchell team employed LNPs containing Mal-PEG lipids conjugated with antibody fragments to deliver chimeric antigen receptor (CAR) mRNA. Animal experiments revealed that these modified LNPs selectively transfected T cells and achieved a B cell depletion rate of up to 90% [92]. According to reports, mRNA nanomedicines can reach the retinal pigment epithelium and Müller glial cells, but they fail to penetrate the neural retina to access photoreceptor cells [93, 94]. This challenge severely limits the application of mRNA nanomedicines in genetic retinal degenerations. To address this issue, a study identified 30 retinal-targeting peptides, which were conjugated to LNPs containing Mal-functionalized PEG lipids [95]. Subsequent evaluation experiments confirmed that the retinal-targeting peptide MH42 had excellent guidance capability, and the resulting MH42-LNPs effectively delivered mRNA to photoreceptor cells in the retinas of mice and rhesus macaques. In addition to the examples mentioned above, there are many similar reports that primarily focus on engineering PEG lipids to achieve organ-specific targeting of mRNA therapeutics. Although PEG lipids offer several benefits, they still have some drawbacks. On the one hand, they may trigger the accelerated blood clearance (ABC) effect, causing LNPs to be cleared more rapidly after multiple administrations. On the other hand, PEG lipids may be associated with side effects of mRNA vaccines, such as allergic reactions [96, 97]. Therefore, when designing mRNA delivery systems, it is essential to consider the pros and cons of PEG lipids comprehensively to balance the targeting and safety of PEG-mediated carriers.

Helper lipids, as crucial components of LNPs, predominantly influence the structural stability, fluidity, and physical integrity of the nanoparticles [98]. In conventional LNP delivery systems, helper lipids typically account for about 10%-20%. These helper lipids modulate LNP characteristics by interacting with other lipid components, which in turn affects the efficiency of drug delivery. 1,2-distearoyl-sn-glycero-3-phosphocholine (DSPC) and 1,2-dioleoyl-sn-glycero-3-phosphoethanolamine (DOPE) are two of the most studied helper lipids, with DSPC having been used in FDA-approved mRNA vaccines and siRNA drugs. The differences in their chemical structures mainly lie in the long carbon chains and head groups, which contribute to slight variations in their biological activity. Research has demonstrated that modulating the type and charge of helper lipids enables mRNA to be targeted to specific organs. Zhang et al. compared the delivery efficacy of DSPC and DOPE in various organs, finding that DSPC-mediated LNPs had over five times higher delivery efficiency in the spleen compared to DOPE-mediated LNPs [99]. In contrast, DOPE-mediated LNPs exhibited superior transfection capability in the liver. The charge carried by helper lipids is also a key factor influencing targeted delivery. According to Benedicto et al., LNPs containing negatively charged helper lipids exhibited higher affinity for the spleen, while neutral phospholipid-mediated LNPs tended to accumulate in the liver [100]. Inspired by cationic lipid structures, Liu et al. introduced ionizable structures into helper lipids with membrane fusion potential, resulting in multi-tailed ionizable phospholipids named iPhos [101]. Compared to commercially available DSPC, iPhos 9A1P9 demonstrated a 965-fold increase in in vivo delivery efficiency. More importantly, mRNA formulations combining iPhos 9A1P9 with dimethyl-dioctadecylammonium (DDAB) enable targeted delivery of cargo to the lungs.

Cholesterol is not only an important component of cell membranes, helping to maintain normal cell function by regulating membrane fluidity and stability, but also a crucial structural lipid in LNPs. Apart from ionizable lipids, it is the second most abundant lipid in LNPs and has a similar regulatory role. Compared to other components in LNPs, research on optimizing the structure of cholesterol for targeted mRNA delivery remains limited. Previous studies have shown that hydroxyl-modified cholesterol can affect its binding kinetics with the Niemann Pick C1 enzyme, thereby altering the endocytic recycling mechanism. Based on this conclusion, Patel and his colleagues developed a library of LNPs containing hydroxycholesterol to improve mRNA delivery [102]. They substituted conventional cholesterol in LNPs with 50% 7α-hydroxycholesterol and observed a twofold increase in mRNA targeting ability to T cells. This finding provides new insights into achieving targeted mRNA delivery through cholesterol modification and identifies potential candidates for cancer immunotherapy. Currently, more researches focus on developing cholesterol analogs to enhance mRNA delivery efficiency. Jung et al. found that 3β[L-histidinamide-carbamoyl] cholesterol conjugated with histidine and cholesterol positively influenced LNP performance, especially in terms of endosomal escape and in vivo delivery efficiency [103]. Considering the potential of natural cholesterol analogs, another study replaced unmodified cholesterol in LNPs with β-sitosterol. The resulting LNPs not only displayed distinct morphological differences compared to conventional LNPs but also achieved greater gene transfection efficiency [104].

#### Polymer-Based Carriers

**Fig. 6:**
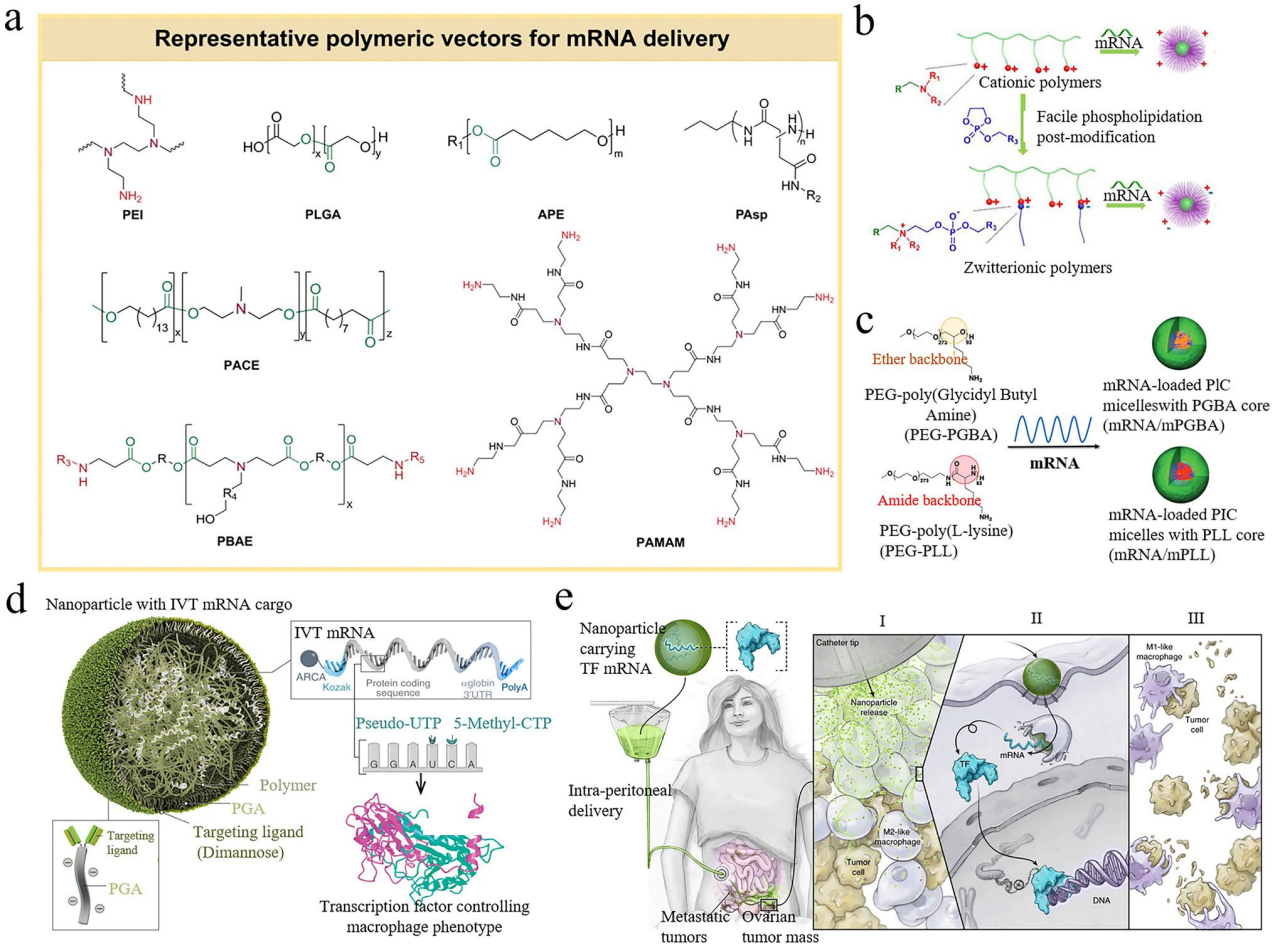
Polymer-based carriers and related targeting strategies. **a** Representative polymer carriers for mRNA delivery. Reproduced with permission from Ref. [105]. Copyright 2024, Royal Society of Chemistry. **b** Zwitterionic polymer carriers facilitated mRNA transport to the spleen and lymph nodes. Reproduced with permission from Ref. [106]. Copyright 2021, American Chemical Society. **c** By modulating the flexibility of the polymer carrier, the bioavailability of mRNA in the lungs was significantly improved. Reproduced with permission from Ref. [111]. Copyright 2020, Wiley − VCH. **d** Schematic diagram of mannose-modified PBAE polymer carrier. **e** Using the PBAE vector modified with mannose, mRNA was efficiently transported to macrophages, leading to the transformation of M2 macrophages into M1 macrophages. (**d, e**) Reproduced with permission from Ref. [114]. Copyright 2019, Springer Nature

Given the inherent fragility of mRNA molecules, developing effective and safe carriers has become increasingly essential. In addition to lipid carriers, polymer carriers are also a key research direction in mRNA delivery due to their diversity and adjustability. Currently, a diverse array of polymer carriers have been developed, including polyethylenimine (PEI), polyamidoamine (PAMAM), poly(lactic-co-glycolic acid) (PLGA), poly(β-amino ester) (PBAE), etc. (Fig. 6a) [105]. Despite the significant progress made, the toxicity, immune response, and mechanisms of degradation and clearance of polymer carriers in vivo still need further optimization to ensure that mRNA can reach target cells and exert its function safely and effectively. This section reviews potential strategies for advancing the development of polymer carriers, such as developing new polymer carriers, optimizing polymer structure or molecular weight, modifying ligands on carrier surfaces, and incorporating lipids or stimulus-responsive units. These strategies are expected to enhance the targeted delivery capabilities of polymer carriers, minimizing non-specific distribution and potential side effects.

By carefully designing new chemical structures, the organ-targeting capabilities of polymer carriers can be significantly improved. As illustrated in Fig. 6b, Liu et al. developed a library of polymers comprising nearly 420 zwitterionic phospholipidized polymers [106]. These zwitterionic phospholipidized polymers, primarily formed by conjugating alkylated dioxaphospholane with cationic polymers, exhibited enhanced membrane fusion properties and targeting capabilities. Impressively, the zwitterionic phosphatidylated polymers exhibited delivery efficiency 39,500 times greater than their parent cationic polymers, which facilitated the targeting of the spleen and lymph nodes following intravenous administration. To deliver payloads to the heart for the treatment of fibrosis, Li et al. proposed a star-shaped polycationic carrier with four arms, mainly composed of ethanolamine-functionalized poly(glycidyl methacrylate)s [107]. The star-shaped polycationic carrier garnered significant attention from researchers, not only for its performance in gene transfection but more importantly, for its therapeutic effects on cardiac fibrosis. Based on the monomers ε-decalactone and ε-caprolactone, Harashima et al. developed an extensive library for mRNA delivery. This library had the potential to avoid liver accumulation and target mRNA to the lungs without the need for targeting ligands [108].

It is important to mention that appropriately adjusting the preparation process, molecular weight, or flexibility of polymer carriers can improve delivery performance and compensate for some shortcomings identified in previous studies. PEI, as a typical cationic polymer carrier, can be categorized into linear and branched types and is extensively studied due to its pulmonary accumulation characteristics. It has been reported that the preparation of linear PEI involves a deacetylation process, and the completeness of this process significantly affects the polymer's physicochemical properties. One study first used commercially available linear PEI with a molecular weight of 25 kDa as the research topic and subjected it to deacetylation. Surprisingly, it was found that the delivery efficiency of the treated carrier in mice increased by 10,000 times, and the lung-specific targeting increased by 1500 times. Based on this discovery, they developed a series of deacetylated PEIs with different molecular weights. Further experimental results confirmed that the deacetylated PEI187 exhibited up to 200 times the lung specificity compared to the deacetylated PEI125, which might be related to the change in the number of protonatable nitrogens [109]. Typically, PEI carriers with high molecular weight are known for their good delivery efficiency. However, the high toxicity of these polymer carriers also limits their clinical application. Therefore, balancing delivery efficiency and toxicity issues is crucial for promoting the development of high molecular weight PEI vectors. For example, Dahlman and colleagues pioneered a PEI_600_-derived polymer carrier characterized by exceptional biocompatibility and efficient delivery, effectively transporting cargo to lung endothelial cells even at remarkably low doses [110]. Another key study proposed an idea that the flexibility of polymer carriers may significantly impact mRNA expression levels. It was demonstrated that compared to the rigid PEG-poly (L-lysine) (PEG-PLL), the highly flexible poly(ethylene glycol)-poly(glycidylbutylamine) (PEG-PGBA) carrier showed strong resistance to enzymatic degradation and enhanced the bioavailability of mRNA in mouse lungs (Fig. 6c) [111].

Ligand modification of polymer carriers, such as sugars, amino acids, peptides, and small molecule drugs, is an attractive strategy to promote targeted delivery. These ligands address the shortcomings of existing polymer carriers, enhance their specific binding ability, and increase the efficacy of mRNA or other cargo at the target site. Chitosan, a kind of natural polysaccharide derived from the deacetylation of chitin, is used in drug delivery systems and biomedical fields due to its excellent biological properties. Considering that cell membranes carry a lot of negative charges, a study utilized positively charged chitosan to modify the polymer PLGA, aiming to enhance the drug uptake level of target cells. The experimental results indicated that these chitosan-modified nanoparticles with high positive charges could accelerate clathrin-mediated endocytosis and increase the likelihood of being captured by pulmonary capillaries [112]. Similar behavior was also observed in another study. The polymer PLGA and chitosan were dissolved in appropriate solvents, and the resulting mixture was then encapsulated into hydroxyapatite to yield HAp/Ch-PLGA nanoparticles. As expected, it was found that HAp/Ch-PLGA nanoparticles were efficiently transferred to the lungs after intravenous administration, while HAp nanoparticles were mainly distributed in the mouse liver [113]. Macrophage mannose receptor 1 (MRC1), also referred to as CD206, exhibits widespread expression on macrophages and has been leveraged for targeted delivery applications. By developing mannose-modified PBAE carriers, mRNA encoding M1-polarizing transcription factor was successfully delivered to tumor-associated macrophages without inducing toxicity (Fig. 6d). Importantly, the targeted mRNA therapy prompted the transformation of M2 macrophages to tumoricidal M1 macrophages, demonstrating strong therapeutic potential in clinical trials for ovarian cancer (Fig. 6e) [114]. In addition to mannose-modified PBAE carriers, other glycosylated polymers have shown promise in targeting tumor-associated cells. For instance, glucose-modified PAMAM synthesized via click chemistry methods can also enhance drug accumulation levels in tumor-associated macrophages and microglial cells, while galactose-modified PAMAM exhibited targeting capabilities toward the tumor microenvironment [115]. Various additional strategies, such as introducing response elements, doping lipid materials, and guiding algorithmic learning, all contribute effectively to enhancing mRNA delivery efficiency and facilitating targeted delivery to specific cells or tissues [116–120].

#### Exosome Carriers

Exosomes are 30–150-nm extracellular vesicles secreted by cells and involved in intercellular molecular communication [121]. Exosomes are considered an ideal drug delivery system for the following reasons: First, the natural biocompatibility of exosomes enables them to effectively fuse with target cells in the body, avoiding the immune rejection reactions that traditional drug delivery systems might cause [122]. Second, exosomes have high stability and can remain in the body for a long time, which is beneficial for the sustained release of drugs [123]. Furthermore, exosomes possess the remarkable ability to cross various biological barriers, such as the BBB, which is crucial for treating hard-to-reach disease sites like those in the nervous system and tumors [124]. In particular, modified exosome-based carriers can achieve targeted delivery to extrahepatic tissues, thereby increasing drug concentration at the target site [125].

**Fig. 7:**
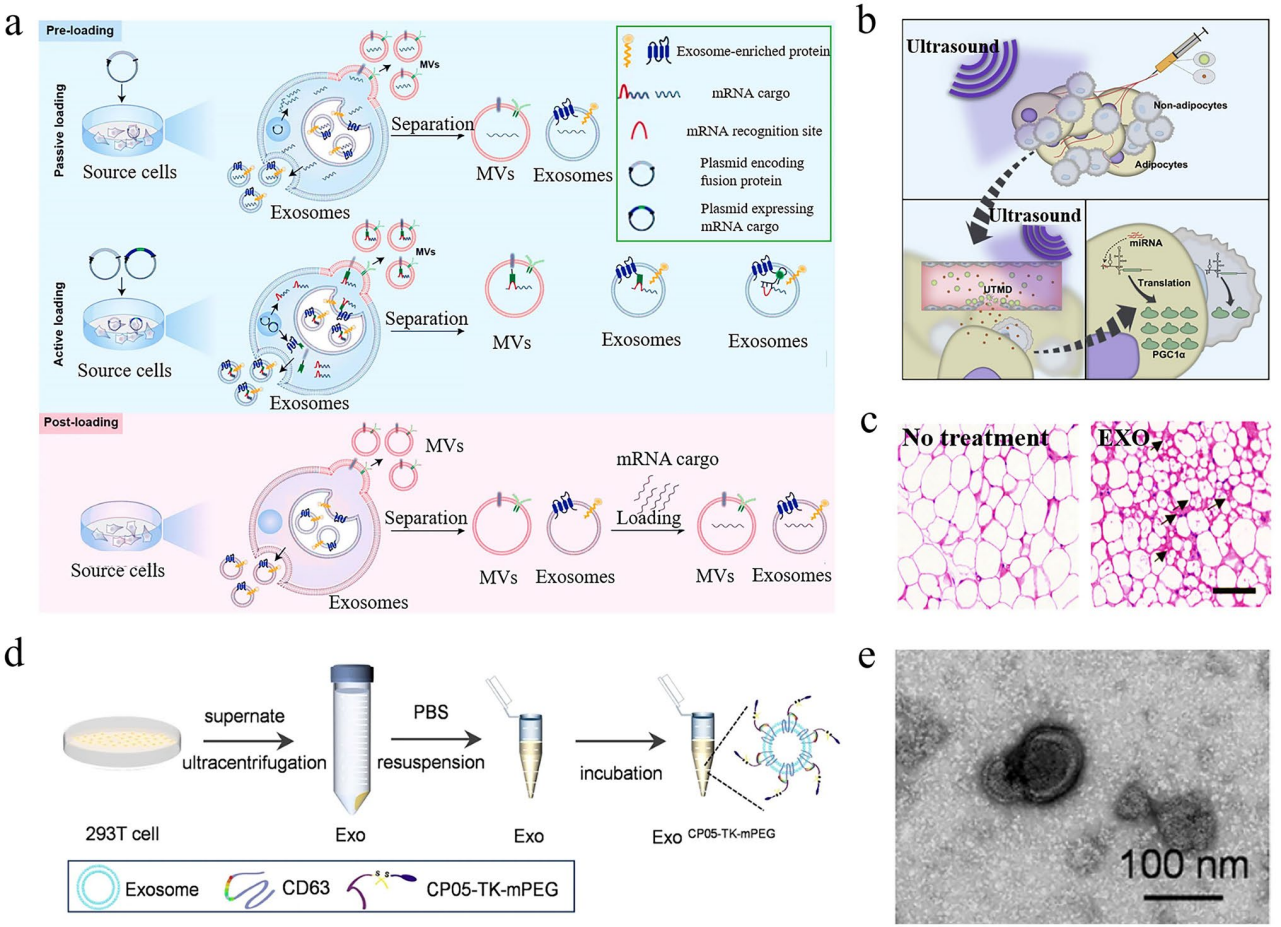
Exosome carriers and related targeting strategies. **a** Techniques for loading cargo into exosomes mainly include pre-loading and post-loading approaches. Reproduced with permission from Ref. [128]. Copyright 2022, MDPI. **b** Under the action of ultrasound, the modified exosomes can be selectively transported to adipocytes for the treatment of obesity. **c** Compared with the untreated group, HE staining showed significant browning in the exosome-treated group. (**b, c**) Reproduced with permission from Ref. [138]. Copyright 2020, Cell Press. **d** Preparation method of CP05-TK-mPEG-modified exosomes. **e** Transmission electron microscopy (TEM) image of CP05-TK-mPEG-modified exosomes. (**d, e**) Reproduced with permission from Ref. [139]. Copyright 2021, Springer Nature

As shown in Fig. 7a, methods for packaging mRNA into exosomes primarily fall into two categories known as pre-loading and post-loading methods [126–128]. For the pre-loading process, plasmids encoding the targeted mRNA are transfected into cells secreting exosomes using non-viral or viral vectors. When cells generate and secrete exosomes, they naturally encapsulate these mRNA within the exosomes. A study transfected cells with DNA encoding low-density lipoprotein receptors, resulting in over a 100-fold increase in mRNA expression levels compared to non-transfected groups [129]. Similar increases were also detected in the secreted exosomes. Post-loading methods, also known as exogenous loading methods, involve co-incubating exosomes with the targeted mRNA under appropriate conditions, allowing the mRNA to be naturally absorbed by the exosomes. Although this method is simple, its efficiency is relatively low. Moreover, advancements in the field have introduced techniques such as commercial reagents or electroporation, which temporarily modify the permeability of exosome membranes, thereby enhancing mRNA loading efficiency [130–133].

Engineering exosomes for targeted delivery is a significant direction in the field of vesicle research. By modifying the surface of exosomes, their targeting performance to specific cells or tissues is improved. For instance, a study used rabies virus glycoprotein to engineer the exosome membrane protein Lamp2b, demonstrating that the modified exosomes could effectively target cargo to ischemic areas of the brain [134]. In a study paralleling previous research, a peptide sequence with adipose-targeting capability (CKGGRAKDC) was introduced to the N-terminus of the exosome membrane protein Lamp2b [135]. This modification not only enabled exosomes to recognize and target adipose tissue more effectively but also improved the delivery efficiency and expression levels of therapeutic mRNA. In addition, Wang and coauthors engineered the surface of exosomes with a high-affinity anti-HER2 scFv antibody, which significantly increased the accumulation of exosomes in target cells, offering a novel approach for targeted breast cancer therapy [136].

To minimize off-target effects, introducing miRNA sequences or using ultrasound assistance is promising alternatives. Reportedly, the internal ribosome entry site (IRES) from the hepatitis C virus can be recognized by liver-specific miRNA-122, thereby initiating mRNA translation in specific tissues [137]. Based on this discovery, a key study by Sun et al. substituted the liver-specific IRES with a fat-specific IRES and encapsulated PGC1α cargo related to energy metabolism in exosomes [138]. With the assistance of ultrasound, it was confirmed that the modified exosomes can be effectively transported to adipose tissue and execute PGC1α mRNA translation, further promoting browning in high-fat diet mice (Fig. 7b, c). Remarkably, Guo et al. developed an advanced exosome-based delivery platform by combining active targeting and ultrasound technology [139]. They first designed a multifunctional stealth coating named CP05-TK-mPEG (Fig. 7d). The peptide component CP05 (CRHSQMTVTSRL) can interact with the exosome surface marker CD63, whereas the PEG component protects the carrier from phagocytosis and prolongs its circulation time in vivo. As shown in Fig. 7e, the CP05-TK-mPEG-engineered exosomes exhibited a roughly spherical shape, with dimensions around 100 nm. Under the action of ultrasound, the Chlorin e6 (Ce6) fused in exosomes rapidly produced reactive oxygen species, leading to the destruction of thioketals and the release of the active component bone morphogenetic protein 7 mRNA. The results indicated that the smart delivery platform offered a new direction for the development of obesity therapies, and the involved targeting strategies may have universal applicability.

#### Other Nanocarriers

By precisely regulating various physical and chemical interactions between nanomedicines and organisms, the targeting specificity, biocompatibility, and transfection efficiency of mRNA carriers are expected to be refined. This intricate regulatory strategy offers limitless possibilities for the design of mRNA carriers, pushing precision medicine to new heights. With a deepening understanding of carrier design concepts, an increasing array of novel nanocarriers, including inorganic nanoparticles, hybrid carriers, biomimetic systems, virus-like particles, etc., are continually emerging. While these emerging carriers exhibit distinct features in design, structure, or composition, they all effectively cross biological barriers and ensure critical roles in targeted therapy. These advancements not only accelerate the development of mRNA therapy but also provide new opportunities for treating complex diseases.

**Fig. 8:**
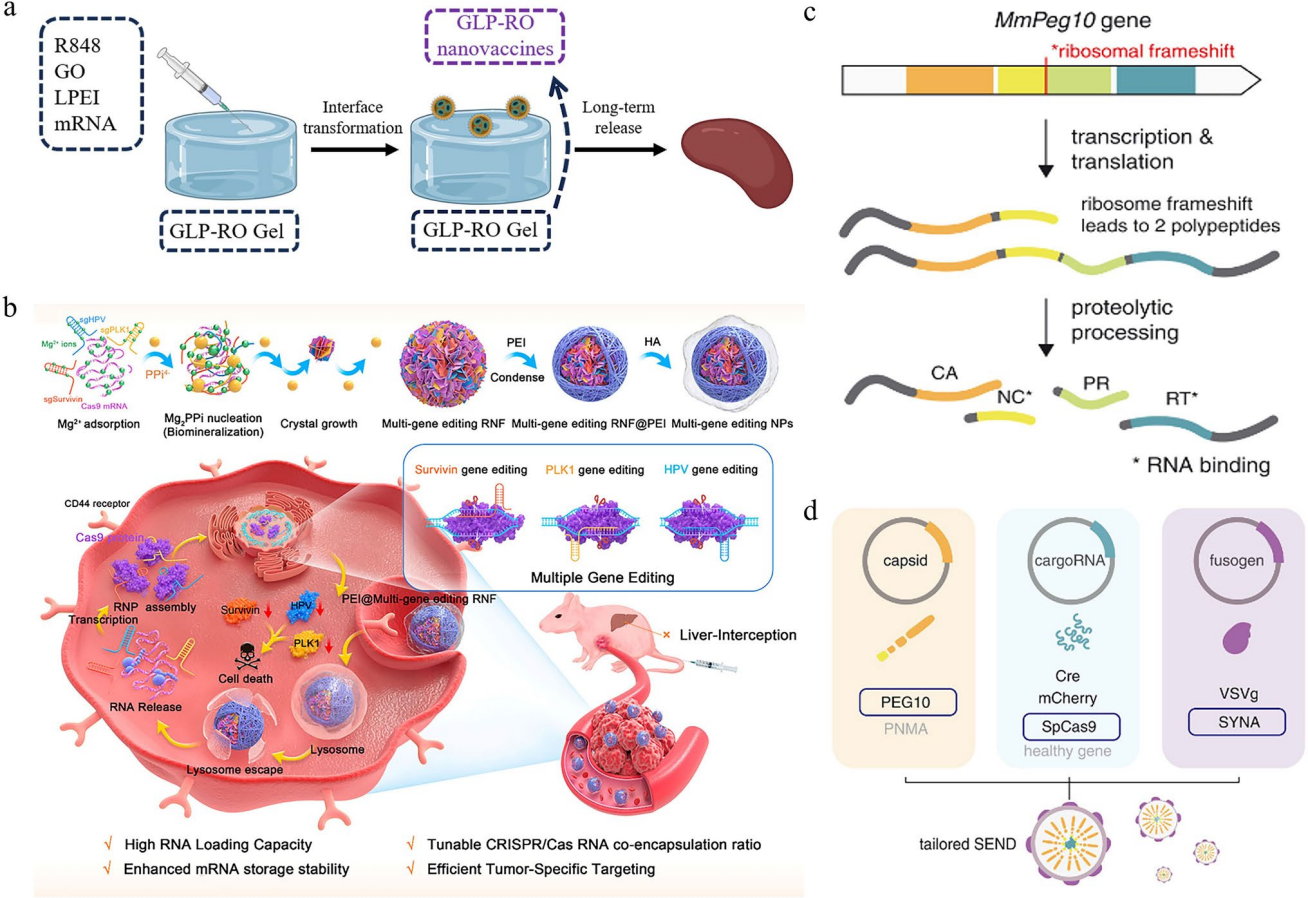
Other nanocarriers and related targeting strategies. **a** A mixed carrier based on GO, PEI, and R484 for targeted mRNA delivery. Reproduced with permission from Ref. [143]. Copyright 2021, American Chemical Society. **b** Preparation and working mechanism of a Mg^2+^-based biomimetic carrier. Reproduced with permission from Ref. [149]. Copyright 2023, American Chemical Society. **c** Four structural domains of the PEG10 gene. **d** Composition of the SEND delivery platform, including cargo RNA, fusogen structure, and PEG10. (**c, d**) Reproduced with permission from Ref. [153]. Copyright 2021, American Association for the Advancement of Science

Graphene oxide (GO) is reported to have unique advantages for drug delivery. Its extensive surface allows for efficient drug loading, and the abundant functional groups on its surface enable drug molecules to bind through physical adsorption, covalent bonding, or electrostatic adsorption [140–142]. The good biocompatibility and tunability of GO further enhance its potential for use in precision drug delivery. As shown in Fig. 8a, a study combined the TLR7/8 agonist resiquimod (R848), the low molecular weight PEI, and the inorganic material GO to encapsulate ovalbumin (OVA) mRNA [143]^.^ The resulting mixture GLP-RO, when injected subcutaneously, targeted the lymph nodes to activate immune cells and demonstrated strong anti-tumor capabilities in a B16-OVA melanoma model. Impressively, these nanomedicines maintained efficacy for at least 30 days, and no significant side effects were observed. Similarly, the integration of lipid materials and polymer carriers also makes it possible for mRNA delivery to other organs. Andretto and his colleagues doped hyaluronic acid as a coating on the surface of cationic lipid-mRNA complexes. The presence of this negatively charged coating fine-tuned the physicochemical properties of the nanoparticles, enhancing stability and promoting mRNA translation in the spleen [144]. In another study, nanomedicines were prepared using a self-assembly strategy by mixing cationic lipid-like compounds, PLGA polymers, and mRNA. It was confirmed that the hybrid carrier can assist in delivering mRNA to functionally deficient prostate cancer cells with extremely low cytotoxicity. Following intravenous administration in a model of PC3 xenograft tumors, the mRNA nanomedicines showed high accumulation levels in mouse tumors and promising therapeutic outcomes [145].

Biomimetic nanocarriers, by mimicking the structure and function of biological systems, demonstrate great potential in mRNA delivery [146]. Typically, these biomimetic nanocarriers can imitate the natural structure such as cell membranes, allowing them to effectively evade recognition and clearance by the immune system during mRNA delivery. In a study on arthritis treatment, plasmids expressing tumor necrosis factor (TNF-α) receptors were pre-transfected into macrophages. The cell membranes of the macrophages, collected by ultracentrifugation, were then used to encapsulate the inner PLGA-mRNA complex. Evidence showed that the resulting biomimetic carriers could quickly target the inflammation sites of arthritis, release therapeutic mRNA, and inhibit the expression of inflammation-related factors after intravenous administration [147]. Another similar study utilized genetic engineering technology to prepare cell membranes with high expression of hemagglutinin. To obtain biomimetic nanoparticles, the PLGA-mRNA complexes were encapsulated in the pre-prepared cell membrane. It was verified that, whether administered locally or systemically, the cell membrane-coated nanocarriers exhibited higher expression levels compared to the non-coated group [148]. Inspired by natural biomineralization, Liang and his colleagues created a Mg^2+^-based biomimetic system for accurate CRISPR/Cas9 RNA delivery (Fig. 8b) [149]. Mg^2+^, as a metal cation, first coordinated with RNA to obtain crystal nuclei. Once the crystals formed, PEI and HA served as external coatings, wrapping around the Mg^2+^-RNA core and manipulating the physical and chemical properties of the carrier. Further investigation demonstrated that the biomimetic delivery system could be stored at 4 °C for at least a month and achieve precise gene editing at tumor sites. With these features in mind, the development of biomimetic nanocarriers presents an effective candidate for targeted mRNA delivery, which is of significant importance in advancing mRNA-based precision medicine.

Virus-like particles (VLPs), which have the shape and size of parent viruses but lack transfective genomes, have garnered significant interest in drug delivery research [150–152]. VLPs effectively protect mRNA from degradation by biological barriers and deliver mRNA to specific cells through their natural cell invasion mechanisms. In 2021, a study published in Science developed a VLP delivery system based on the mammalian retroviral-like protein PEG10, called SEND [153]. As shown in Fig. 8c, d, the SEND delivery platform was primarily composed of cargo RNA, a fusogen, and PEG10 with four structural domains, featuring modular and customizable characteristics. By using the SEND platform, gene editing tools were successfully delivered to human cells. Based on this study, Li et al. further demonstrated that PEG10-based VLPs can also be applied for treating eye diseases [154]. Impressively, a large quantity of PEG10-based VLPs was delivered to retinal pigment epithelial cells through retinal administration, resulting in high mRNA expression levels. Besides, the VLPs exhibited lower immunogenicity compared to other traditional lentiviral vectors, largely because PEG10 was an endogenous human protein. Leveraging five plasmids, such as pMD.2G, pRSV-REV, pMDlg/pRRE-D64V, pCMV-spike-6 × MS2, and pMS2M-PH-gagpol-D64V, Yin et al. developed dendritic cell-targeted VLP carriers. Compared to LNP formulations at the same dose, mRNA vaccines based on VLPs induced stronger cellular and humoral immunity in mice and offered longer-lasting protection. Furthermore, the VLP carriers were extended for use in herpes simplex virus vaccines, which also achieve ideal antiviral effects [155]. With the continuous advancement of research, breakthroughs in emerging nanocarrier technologies will broaden the application prospects of mRNA-targeted therapies, facilitating the realization of personalized medicine.

## Targeting Mechanisms and Biological Barriers of mRNA Nanomedicines

### Targeting Mechanisms

Once mRNA nanomedicines are in the bloodstream, a complex array of biological processes collectively influences their ultimate fate within the body. To maximize the efficacy and safety of mRNA therapy, the key lies in precisely delivering mRNA nanomedicines to specific target organs or target cells. The targeting mechanisms of mRNA nanomedicines mainly involve three levels: passive targeting, endogenous targeting, and active targeting [156–158]. When designing targeted delivery systems, these mechanisms can be used individually or in combination to precisely control the localization of nanoparticles within the body, ensuring the maximization of drug delivery efficiency and therapeutic effect.

#### Passive Targeting

**Fig. 9:**
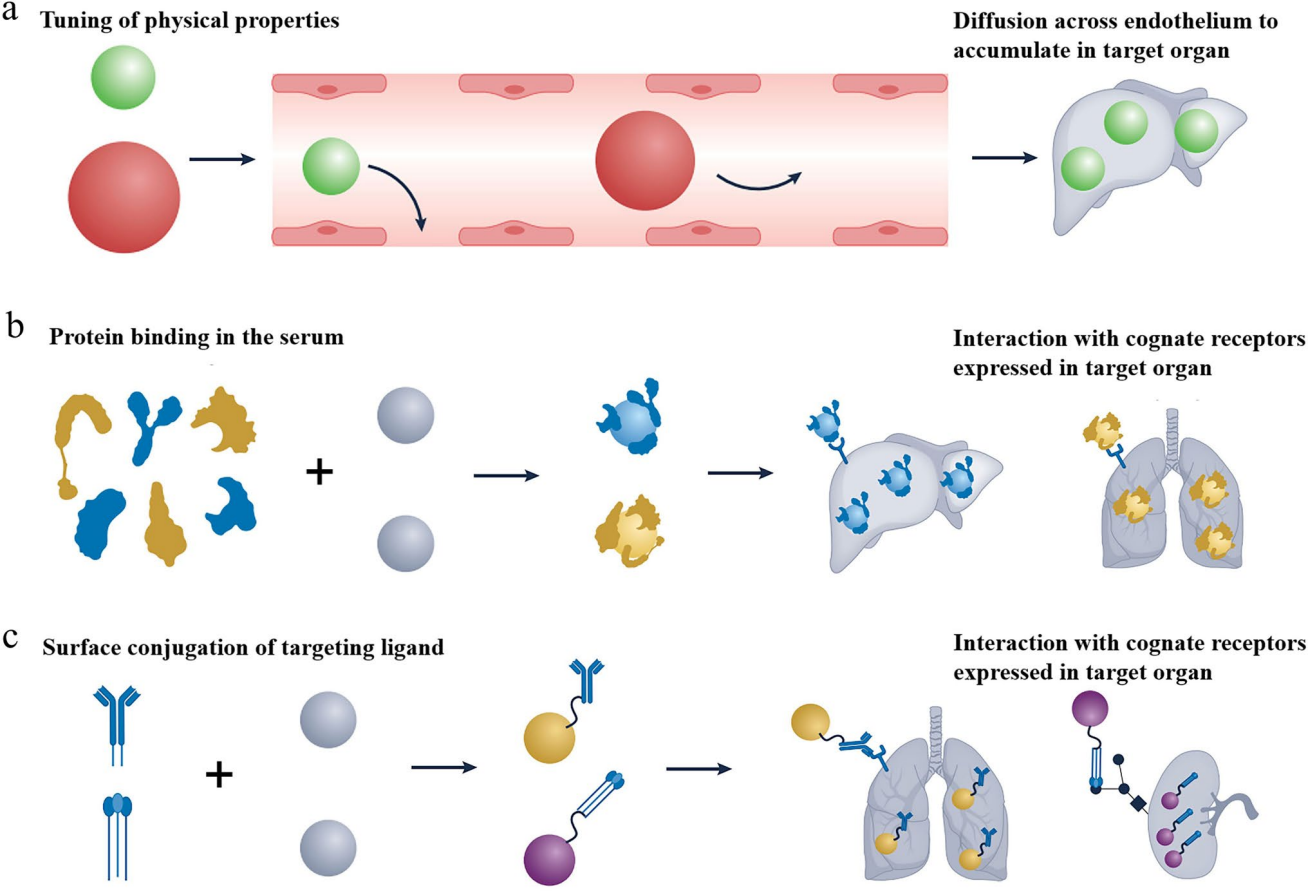
Mechanisms of passive targeting, endogenous targeting, and active targeting. **a** Mechanism of passive targeting. Passive targeting is closely related to the physicochemical characteristics of nanoparticles, including size, morphology, surface charge, etc. **b** Mechanism of endogenous targeting. Endogenous targeting is a natural transport mechanism within organisms. Once mRNA nanomedicines enter the body, they bind with specific proteins in the plasma and are subsequently transported to target organs. **c** Mechanism of active targeting. Active targeting refers to specific biomolecule or ligand modifications on delivery systems, which endow mRNA nanomedicines with the ability to target specific organs without causing unnecessary impact on surrounding healthy tissues. Reproduced with permission from Ref. [156]. Copyright 2023, Springer Nature

Passive targeting of mRNA nanomedicines leverages the inherent characteristics of drug carriers to enhance drug accumulation in specific tissues or cells, without relying on specific biological molecules or ligands for recognition [159]. By adjusting nanomedicine characteristics like size, morphology, surface charge, and other parameters, passive targeting allows drugs to naturally distribute within the body (Fig. 9a) [160]. Additionally, the physiological structural characteristics of target organs are closely associated with passive targeting, influencing the distribution and retention of nanoparticles in vivo.

For instance, nanoparticles with diameters between 100 and 150 nm are considered critical for accumulation in the liver [161]. Within this size range, nanoparticles are readily recognized by the liver's reticuloendothelial system, while they can also penetrate blood vessels to reach tumor and inflammatory sites. In contrast, particles larger than 7 µm are filtered and trapped by the lung's capillary network after intravenous injection. This occurs because the typical diameter of pulmonary capillaries ranges from 5 to 10 µm, which restricts the passage of larger particles. In addition to size, the shape of nanomedicines is crucial for effective passive targeting. Spherical nanoparticles, due to their symmetrical properties, typically exhibit uniform kinetic behavior in blood circulation, facilitating higher accumulation rates in the liver or other organs. In comparison, rod-shaped nanoparticles experience significant changes in their blood circulation time, as they may encounter more obstacles when passing through narrow capillaries. The surface charge of nanoparticles significantly impacts their behavior and ultimate fate within the body [162]. While positively charged nanoparticles bind more easily to cell membranes, they are also more prone to clearance by the immune system. Negatively charged or neutral nanoparticles tend to remain in the bloodstream for a longer duration.

#### Endogenous Targeting

Endogenous targeting is a special form of passive targeting that leverages natural mechanisms or signaling pathways within cells to achieve targeted delivery of therapeutic drugs or genes. This approach typically relies on molecular tools such as small molecules, nucleic acids, or proteins that exist within the body, directly intervening or modulating intracellular signaling pathways [22]. Although endogenous targeting does not require additional targeting ligands, it is crucial to consider the components transported by endogenous proteins during the drug design process.

As shown in Fig. 9b, mRNA formulations largely bind to various biomolecules within the body following intravenous administration, forming a specific “biomolecular corona,” particularly a “protein corona” formed by binding with proteins in the blood [163, 164]. Notably, when the researchers deeply explore the fate between mRNA nanomedicines and protein corona in vivo, they find that protein corona has a dual effect on the targeting effect of mRNA drugs. It is well known that controlling the adsorption of the protein corona is challenging, and this protein corona may mask nanomedicines, potentially causing targeting abnormalities [165]. However, some evidence suggests that the protein corona in the blood can also be utilized and regulated to achieve selective targeting. For instance, apolipoprotein E (ApoE) is an important lipoprotein primarily synthesized and secreted by liver cells, essential for lipid metabolism and transport. The combined characteristics of ApoE and mRNA formulations facilitate targeted delivery to the liver [166]. Furthermore, another study has demonstrated that the elasticity of nanoparticles significantly influences the types of protein corona and the lifespan of the nanoparticles in the bloodstream [167]. Together, the complex interaction reveals that protein corona not only affects the stability and circulation time of nanomedicines but also influences their targeting and therapeutic efficacy.

#### Active Targeting

Active targeting is an advanced nanomedicine delivery technology that involves engineering the nanocarriers with specific biomolecules, such as antibodies, peptides, or small molecule ligands, enabling them to specifically identify and interact with receptors on the surface of target cells (Fig. 9c) [168–170]. By using ligand–receptor interactions, this process allows the drug to be delivered more precisely to the diseased areas. Compared to passive targeting, active targeting significantly enhances drug accumulation in specific cells or tissues and reduces systemic toxicity and side effects.

In preclinical studies, mRNA nanomedicines with active targeting capabilities have demonstrated significant potential in areas such as pulmonary diseases, brain disorders, and bone marrow diseases [171]. Parhiz and colleagues modified the surface of PEG lipids with anti-CD31, which allowed the resulting LNP nanomedicines to be effectively delivered to the lungs, independently of ApoE [172]. Using anti-vascular cell adhesion molecule-1 (VCAM-1) modification on nanocarriers, the engineered mRNA formulations can efficiently cross the BBB, facilitating de novo translation of mRNA cargo within the brain [29]. However, active targeting technology still faces several challenges [173–175]. On the one hand, the manufacturing process is complex and costly, and modified biomolecules may be unstable and provoke immune reactions. On the other hand, fluctuations in receptor expression levels also affect the consistency of therapeutic outcomes. These factors can lead to variable efficacy and safety profiles, necessitating ongoing refinement of targeting strategies and production methods to improve overall therapeutic performance.

### Biological Barriers

As an emerging therapeutic approach, mRNA nanomedicines exhibit tremendous potential. However, for their clinical application to be realized, mRNA nanomedicines must overcome a series of biological barriers, including the circulatory barrier, endothelial barrier, and endosomal barrier. The circulatory barrier pertains to the stability of nanomedicines and their distribution in the bloodstream. The endothelial barrier primarily concerns how nanomedicines traverse blood vessel walls to reach target tissues. By contrast, the endosomal barrier mainly involves intracellular transport and release mechanisms. These barriers collectively affect the therapeutic efficacy and safety of mRNA nanomedicines. A thorough understanding and effective addressing of these barriers are crucial for the development of efficient mRNA nanomedicines.

#### Circulation Barrier

In the development of mRNA nanomedicines, a significant challenge is ensuring that these drugs successfully overcome circulation barriers and effectively enter desired cells while avoiding bystander cells. This stringent requirement highlights the difficulty in developing precisely targeted mRNA nanomedicines. A widely known strategy involves incorporating PEG-derived lipids on the surface of nanocarriers [176]. It has been reported that the addition of PEGylated lipids can provide a “stealth” coating to evade immune system recognition, reduce non-specific protein adsorption and clearance rates in the blood, and extend the drug's half-life in vivo [177]. One study coupled the ligand cyclic arginine-glycine-aspartic acid (cRGD) with PEG lipids, and further investigation confirmed that the engineered lipids markedly improved the circulation duration and stability of the nanomedicine [178]. Interestingly, a mere twofold increase in PEGylation density resulted in a tenfold increase in mRNA expression within the tumor. Another study incorporated the thermoresponsive material poly(N-isopropylacrylamide) (PNIPAM) onto the surface of cRGD-modified PEG-polylysine (PLys), forming polymeric micelles with a stable thermodynamic structure that effectively prevented nuclease degradation [179]. In the bloodstream, the modified nanoformulations exhibited a longer circulation time compared to the unmodified ones.

However, despite the significant drug delivery advantages conferred by PEGylation, excessive use of PEG lipids carries the risk of side effects. Firstly, the presence of PEG has the potential to trigger a phenomenon called ABC. ABC refers to the rapid clearance of PEGylated nanoparticles upon repeated injection due to the body's production of anti-PEG antibodies, which significantly decreases therapeutic efficacy [180–182]. Besides, this immune response may induce allergic reactions, ranging from mild rashes to severe anaphylactic shock, posing a potential threat to patient safety [183]. Some studies have shown that excessive PEGylation enables nanoparticles to form a “stealth layer” near the target cells, which may reduce the interaction between the nanoparticles and target cells, thereby affecting their targeting ability and drug release efficiency. Lastly, the metabolism of PEG lipids also warrants additional attention. As a synthetic polymer, PEG lipids are degraded and excreted slowly in vivo, and the long-term accumulation could pose risks of hepatotoxicity and nephrotoxicity [184–186]. When designing mRNA nanomedicines, it is crucial to balance the bioavailability provided by PEGylation with its potential side effects.

In addition, engineered nanocarriers using natural cell membranes or biomimetic materials are expected to overcome circulation barriers and achieve organ-specific targeted delivery. Notable progress has been made with calcium carbonate nanoparticles modified with cancer cell membranes for mRNA delivery to the brain [187]. The results demonstrated that these modified nanoparticles rapidly traversed the BBB following intravenous injection and released interleukin-12 (IL-12) mRNA under ultrasound control. In another typical example, Dong et al. established a biomimetic nanoparticle library using phospholipids and glycolipid derivatives [188]. Through continuous screening and optimization, the bionic delivery system PL1 can effectively bypass the circulation barrier and deliver mRNA to T cells.

#### Endothelial Barrier

The endothelial barrier is primarily composed of endothelial cells lining the blood vessel walls, which form a continuous monolayer structure. This barrier acts as a selective permeability barrier, controlling the passage of ions, nutrients, and other molecules between the bloodstream and surrounding tissues [189–191]. Maintaining the integrity of this barrier is crucial for regulating the transport of substances into and out of blood vessels, thereby ensuring vascular homeostasis and proper tissue function. In the context of nanomedicine delivery, the endothelial barrier is a critical hurdle. Nanoparticles designed for therapeutic purposes must effectively cross the barrier to reach target tissues or cells within the body. However, the endothelial barrier's tight junctions and regulatory mechanisms pose significant challenges for the efficient delivery of macromolecular drugs and nanoparticles [192–194]. The barrier limits the free penetration of larger molecules, requiring innovative strategies to enhance transport efficiency.

To overcome these challenges, researchers have devised a range of strategies to enhance nanomedicine delivery efficiency. One approach involves engineering nanomedicines with specific surface modifications to facilitate their transendothelial passage and enable selective binding to target cells [195]. Furthermore, manipulating the shape, size, and surface charge of nanomedicines to alter their permeability through endothelial cells is another common strategy [196]. Smaller nanomedicines generally penetrate the endothelial barrier more easily than larger ones, and nanomedicines with positively charged surfaces tend to enhance cellular uptake by binding more readily to negatively charged cell membranes. Currently, some studies have demonstrated that nanomaterials with photothermal properties can convert light energy into heat, which induces a localized temperature increase [197]. This temporarily enhances the permeability of endothelial cells, further improving the penetration and delivery of nanomedicines. It is essential to mention that endothelial cells are connected in various ways, including tight junctions, adherent junctions, and gap junctions. These connections not only provide strong adhesion between cells but also form tight physical barriers that prevent nanoparticles from freely passing among cells. However, through ultrasound therapy, these tight junctions can be temporarily opened, which increases intercellular permeability and facilitates more effective delivery of drugs to target sites [139].

Notably, the function and structure of the endothelial barrier undergo significant changes in different disease states. For example, in tumor tissues, the endothelial barrier of blood vessels frequently shows incomplete structure and heightened permeability, known as the enhanced permeability and retention (EPR) effect [198]. This phenomenon enables the specific accumulation of nanomedicines at tumor sites, which has a positive impact on tumor therapy. In certain inflammatory or cardiovascular diseases, the integrity of the endothelial barrier may also be compromised, leading to variations in treatment outcomes [199–201]. Therefore, a deep understanding of the changes in the endothelial barrier under disease conditions is crucial for designing and optimizing efficient delivery systems. Such understanding not only improves the targeting and efficacy of drugs but also advances personalized medicine by providing more precise treatment strategies for patients.

#### Endosomal Barrier

**Fig. 10:**
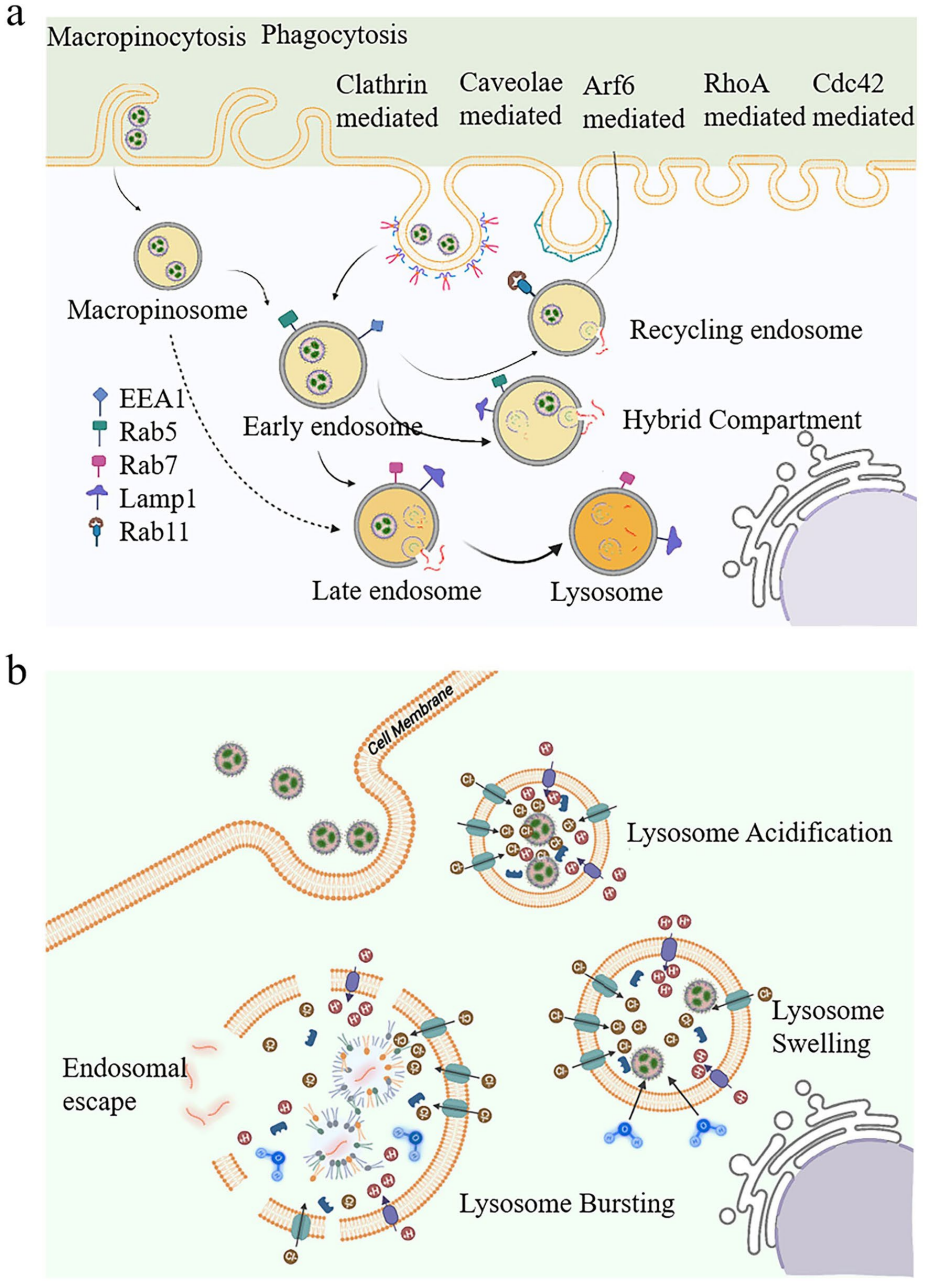
Introduction to the endosomal barrier. **a** Typically, LNPs are taken up through clathrin-mediated endocytosis and macropinocytosis and then transferred to early endosomes. Unfortunately, most nanomedicines struggle to escape the endosomes and are ultimately cleared. **b** Diagram illustrating the escape of LNPs from endosomes by the proton sponge effect. In acidic environments, LNPs activate proton pumps and cause a large influx of protons, leading to increased osmotic pressure and rupture of the endosomal membrane. Reproduced with permission from Ref. [205]. Copyright 2024, National Academy of Sciences

The endosomal barrier of nanomedicines refers to a series of complex obstacles that nanomedicines encounter after entering the cell [202–204]. Once inside the cell, nanomedicines are typically captured by endosomes through endocytosis, forming endosomal vesicles. With the maturation of endosomes, the internal pH gradually decreases, and enzyme activity significantly increases. This acidic environment and enzyme activation may lead to the degradation or inactivation of nanomedicines, severely impacting their therapeutic efficacy (Fig. 10a) [205]. For nanomedicines to exert their intended pharmacological effects, they must successfully escape from the endosome into the cytoplasm. This escape is critical because it enables the nanomedicines to avoid degradation within the acidic endosomal environment and deliver their therapeutic payloads intact. Based on these features, enhancing the efficiency of endosomal escape is the key to developing effective nanomedicines.

Reportedly, various approaches have been developed to promote the escape of nanomedicines from endosomes into the cytoplasm. A frequently used strategy employs pH-sensitive materials that react to the acidic environment inside endosomes. These materials undergo structural changes or disassembly, triggering the release of encapsulated drugs or therapeutic agents. For instance, LNPs derived from ionizable lipids are designed to remain stable at physiological pH but become protonated and destabilize under acidic conditions within endosomes (Fig. 10b) [206]. This destabilization facilitates the release of their cargo and boosts the efficiency of drug delivery into the cytoplasm. Another strategy employs membrane-active agents, such as membrane lytic peptides, which destabilize the endosomal membrane and promote the release of nanomedicines. Sakamoto et al. designed a membrane lytic peptide containing aminoadipic acid residues, which has been shown to effectively disrupt membrane stability [207]. In addition, methods like photothermal or photochemical treatments have been explored to induce endosomal escape. These methods utilize external stimuli such as light to generate heat or reactive oxygen species, causing endosomal membrane disruption and subsequent release of therapeutic payloads [208].

Along with the previously mentioned strategies, recent years have witnessed the emergence of novel endosomal escape methods based on nanotechnology. For example, some researchers have developed a combination of magnetic nanoparticles and external magnetic fields to manipulate the positioning of nanomedicines within cells [209, 210]. These magnetic nanoparticles can generate mechanical force when exposed to a magnetic field, resulting in significant damage to the endosomal membrane [211, 212]. Such innovative techniques not only provide more efficient drug delivery pathways but also improve therapeutic outcomes.

## mRNA-Based Targeted Strategies

mRNA targeting therapy represents a revolutionary breakthrough in the medical field, aiming to precisely deliver therapeutic genetic information to specific organs or sites. However, achieving this goal is not an easy task. In this cutting-edge treatment, scientists face challenges in overcoming technical obstacles such as mRNA stability and immunogenicity, as well as addressing the complexity of organ-specific delivery. To tackle these challenges, they are exploring strategies such as developing novel ionizable lipids, altering the composition of LNPs, conducting ligand modifications on carrier surfaces, changing delivery methods, etc. These advancements offer new hope for the treatment of liver, lung, heart, brain, and rare diseases, potentially enhancing patients' quality of life and extending their lifespan. Therefore, this section focuses on achieving organ targeting of mRNA through advanced strategies.

### Liver Targeting

**Fig. 11:**
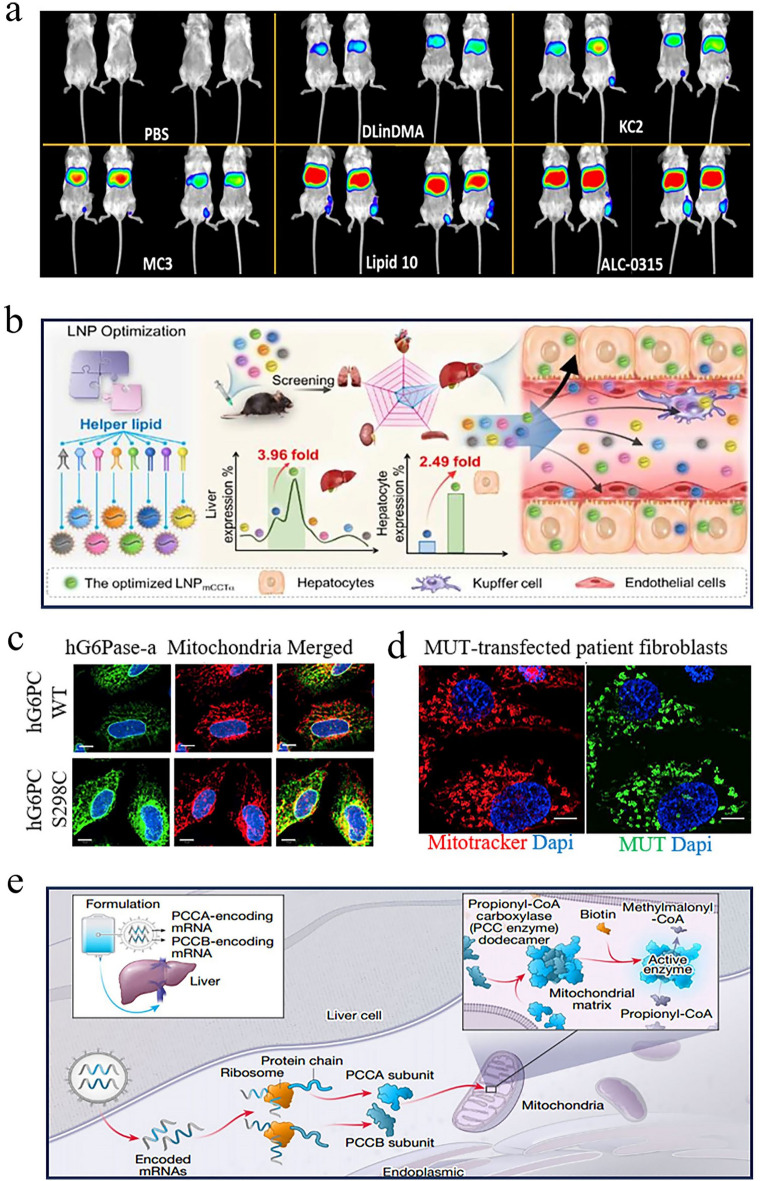
Targeted mRNA therapy for liver diseases. **a** Studies indicated that most ionizable lipid-derived LNPs possessed liver-targeting capabilities. Reproduced with permission from Ref. [214]. Copyright 2023, Wiley − VCH. **b** By optimizing the helper lipids in the LNPs, the expression level of mRNA in the liver increased by 3.96 times. Reproduced with permission from Ref. [215]. Copyright 2024, American Chemical Society. **c** Compared to the hG6PC WT mRNA group, the variant hG6PC S298C mRNA exhibited higher protein expression levels, highlighting the importance of sequence optimization. Reproduced with permission from Ref. [221]. Copyright 2021, Springer Nature. **d** After receiving hMUT mRNA transfection, fibroblasts from patients successfully expressed hMUT, while also exhibiting stable colocalization with mitochondrial markers. Reproduced with permission from Ref. [222]. Copyright 2017, Cell Press. **e** Schematic diagram of mRNA expressing PCCA and PCCB for the treatment of propionic acidemia. Reproduced with permission from Ref. [223]. Copyright 2024, Springer Nature

Generally, most LNP-mRNA nanomedicines tend to accumulate in the liver rather than other organs after intravenous administration, primarily due to several factors [213]. (1) As one of the organs rich in blood vessels in the human body, the liver receives a large volume of blood, facilitating the exposure of nanoparticles to liver cells. (2) When LNP-mRNA nanomedicines enter the bloodstream, the gradual separation of PEG lipids from the surface of LNPs allows ApoE to bind to LNPs. This interaction enhances the uptake of LNPs by low-density lipoprotein receptors (LDL-R). (3) The liver harbors abundant macrophages, which play crucial roles in immune surveillance and defense. These cells are capable of identifying and engulfing nanoparticles, pathogenic microorganisms, cell debris, and other substances from the bloodstream. In a typical example, Lam et al. utilized LNPs derived from different ionizable lipids to deliver firefly luciferase (Fluc) mRNA and observe their expression sites in vivo [214]*.* As expected, the liver region of all mice in the experimental groups consistently exhibited the strongest biological signals, indicating that LNP-mRNA nanomedicines primarily accumulate in the liver (Fig. 11a).

Given this natural targeting capability, mRNA nanomedicines are particularly suitable for treating different types of liver diseases. There is abundant evidence indicating that this therapeutic approach has achieved encouraging results not only in addressing common conditions such as fatty liver, hepatitis, liver fibrosis, and hepatocellular carcinoma (HCC) but also in treating some rare liver diseases. A study attempted to utilize a combination of hepatocyte growth factor and epidermal growth factor mRNA-LNP to promote liver regeneration in models of fatty liver disease. Researchers found that protein expression persisted in the liver for three days after injection of Fluc mRNA formulations, confirming the liver as the primary target organ for mRNA nanomedicines. Importantly, these nanomedicines almost transfected all endothelial cells, hepatocytes, and Kupffer cell subpopulations, accelerating liver function recovery in a chronic liver injury mouse model. Another study aimed to improve the delivery efficiency of mRNA in the liver by screening helper lipids [215]. Using 1-Palmitoyl-2-oleoyl-sn-glycero-3-phosphocholine (POPC) as a helper lipid, the expression level of mRNA in the liver increased by 3.96 times, which showed great promise in the management of metabolic dysfunction-associated fatty liver disease (Fig. 11b). In the case of hepatitis, the use of mRNA to encode anti-HBsAg antibodies delivered through LNPs significantly inhibited HBsAg expression in chronic hepatitis B. Furthermore, mRNA therapies expressing hepatocyte nuclear factor alpha (HNF4A) and ATP-binding cassette 4 (ABCB4) were effective in reversing liver fibrosis. HCC, the most prevalent primary liver cancer, poses a major public health threat due to its aggressive nature and high mortality rate. Research confirmed that IL-12 mRNA formulations possessed the precise targeting ability toward the liver, increased the production of interferon-γ, activated immune responses, and slowed down the progression of HCC, with no significant toxicity observed.

In addition to investigating the therapeutic effects of mRNA therapy on various liver diseases, the current research trend is focused on targeting specific cell populations within the liver to achieve more precise treatment outcomes. This includes targeting hepatocytes, sinusoidal endothelial cells (SECs), Kupffer cells, and other liver-related cell types. Hepatocytes are usually associated with rare diseases related to metabolic and endocrine dysfunction, while SECs are closely linked to chronic liver diseases [216, 217]. A study demonstrated that LNPs derived from MC3 and cKK-E12 interacted with various cell types within the liver microenvironment [218]. This discovery was very important because it was commonly believed that mRNA delivery primarily occurred in hepatocytes. However, experiments showed that these LNPs not only effectively interacted with hepatocytes but also involved other key cell types, such as SECs and Kupffer cells. Usually, LNPs can be easily delivered to these cells through an ApoE-dependent cell uptake mechanism, but the delivery efficiency in SECs was significantly lower than in hepatocytes [219]. One study demonstrated that adjusting the size of LNPs, altering the content of PEG lipids, and introducing targeting ligands could improve mRNA delivery to various liver cell types [220]. For example, increasing the content of PEG lipids enhanced targeted delivery to hepatocytes, while mannitol-modified LNP delivery systems allowed more mRNA to be selectively delivered to SECs. By focusing on specific cell types, mRNA nanomedicines can deliver therapeutic information more effectively, minimizing impacts on other tissues and maximizing treatment efficacy.

Up to date, many mRNA therapies have entered clinical trial stages in the field of liver disease treatment. These trials are designed to evaluate the safety, effectiveness, and influence on disease progression of mRNA nanomedicines. Encouragingly, certain initial clinical trials have demonstrated promising outcomes. Glycogen storage disease type 1a (GSD1a) is a rare genetic disorder that affects the metabolism of glycogen in the body, primarily caused by a deficiency of glucose-6-phosphatase-alpha (G6Pase-α). This abnormal accumulation of glycogen can lead to a range of symptoms, including hepatomegaly, hypoglycemia, muscle weakness, and growth retardation. Reportedly, Moderna developed mRNA encoding G6Pase-α and delivered it to the liver via LNPs [221]. To enhance the expression level of G6Pase-α, researchers designed a total of 20 variants of G6Pase-α mRNA, with hG6PC S298C mRNA demonstrating the most outstanding performance (Fig. 11c). Following repeated administrations, fasting blood glucose levels in model mice were restored, and several hepatic and serum biochemical markers associated with GSD1a returned to normal. Currently, this study has entered Phase I/II clinical trials, focusing on evaluating the efficacy and safety of GSD1a treatment. Similarly, mRNA-3705 and mRNA-3927 have entered clinical trials for treating methylmalonic acidemia (MMA) and propionic acidemia, respectively. MMA is a rare genetic metabolic disorder characterized by the inability of the body to properly process certain proteins and fats. This disorder is caused by a deficiency of an enzyme called human methylmalonyl-CoA mutase (hMUT), which is essential for breaking down certain amino acids and fats into energy. As shown in Fig. 11d, the stable colocalization of mitochondrial markers and hMUT in all transfected cells indicated that hMUT mRNA effectively guided the biosynthesis of active enzymes [222]. Multiple administrations of hMUT mRNA formulations significantly reduced plasma methylmalonic acid levels, largely correlating with hMUT expression in the liver. In 2024, Moderna reported the interim clinical results of mRNA-3927, which was delivered to the liver for the expression of propionyl-coenzyme A carboxylase α or β (PCCA or PCCB) subunits to participate in the conversion of propionic acid (Fig. 11e). In this clinical trial, a total of 346 doses of mRNA-3927 were administered, and no dose-limiting toxicities occurred [223]. With further research and clinical trials underway, we can expect to witness more successful cases of mRNA therapy in the treatment of liver diseases, providing patients with more effective treatment options.

### Lung Targeting

The lung is a key component of the human respiratory system, primarily responsible for inhaling oxygen and transporting it into the bloodstream, while simultaneously expelling carbon dioxide from the blood into the external environment. Due to the lung's unique physiological structure, inhalation administration has become a feasible option. The large surface area of alveoli and abundant vascular network provide an effective absorption pathway for inhaled medications, allowing drugs to rapidly enter the bloodstream and achieve systemic bioavailability. Simultaneously, this review also addresses the intravenous delivery of mRNA to the lungs, though this approach encounters additional biological challenges within the body. For lung targeting, delivery systems must avoid accumulation and clearance by the liver, bind to endothelial cells, and penetrate the basement membrane to reach various cells within the lungs. Furthermore, the immune system of the lungs is also a crucial factor, capable of identifying and clearing pathogens and foreign substances in the body, thus maintaining the health of the respiratory tract.

**Fig. 12:**
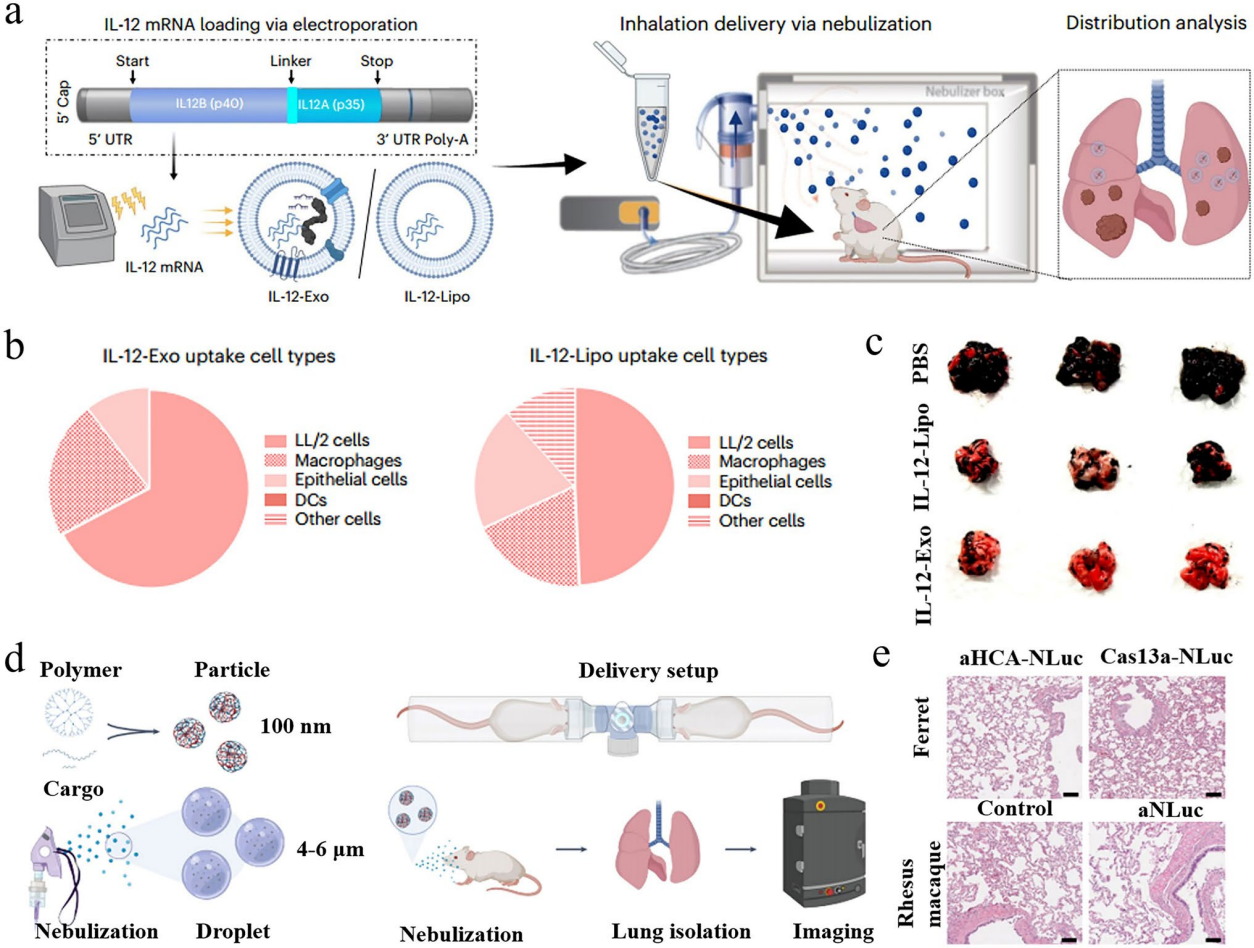
Inhalation administration achieves lung delivery of mRNA. **a** IL-12 mRNA was encapsulated by exosomes or liposomes, followed by inhalation administration to treat lung cancer. **b** The data showed the amount of IL-12-Exo and IL-12-Lipo taken up by different types of cells. **c** Orthotopic lung tumors in the IL-12-Exo group showed significantly greater improvement compared to those in the IL-12-Lipo and the PBS groups. (**a, c**) Reproduced with permission from Ref. [224]. Copyright 2024, Springer Nature. **d** Preparation, nebulization, and inhalation administration of polymer P76-mediated nanomedicines. **e** Lung staining from ferrets and macaques showed that the polymer-based mRNA therapy had no toxicity. (**d, e**) Reproduced with permission from Ref. [225]. Copyright 2023, Springer Nature

Inhalation administration is a significant method for facilitating mRNA delivery to the lungs. This method involves converting mRNA nanoparticle formulations into fine particles using an atomizer and then inhaling them into the respiratory tract. Through inhalation administration, Liu et al. successfully directed the delivery of exosome-packaged IL-12 mRNA (IL-12-Exo) to the lungs, achieving effective treatment for lung cancer **(**Fig. 12a**)** [224]. This inhalation-based delivery method harnessed the natural properties of extracellular vesicles to promote the absorption and utilization of mRNA in the lungs. Importantly, IL-12-Exo exhibited higher uptake efficiency in LL/2 cell lines compared to IL-12 mRNA packaged in traditional liposome carriers (IL-12-Lipo), suggesting the potential of IL-12-Exo in lung diseases **(**Fig. 12b**)**. Further animal model studies also supported this view, as IL-12-Exo treatment significantly inhibited tumor growth **(**Fig. 12c**).** Another study screened 166 polymer nanoparticle formulations and found that polymer P76 had higher delivery efficiency [225]. Using nebulization administration, the polymer P76-mediated nanomedicines induced stronger antibody levels in a mouse model challenged with SARS-CoV-2 while exhibiting good tolerance **(**Fig. 12d**)**. Similar experimental results were confirmed in other animal models, such as ferrets, hammers, rhesus macaques, etc. **(**Fig. 12e**)**. In addition, inhalation administration also faces unique challenges, such as the stability of mRNA formulations and the biological barrier [226–228]. To address these challenges, Jiang et al. significantly improved the stability of mRNA formulations during nebulization by altering the nebulization buffer and adding branched polymer excipients [229]. Furthermore, by designing a novel lipid library, researchers achieved efficient penetration of the biological barrier. In a key study, Li et al. synthesized a combinatorial library of biodegradable ionizable lipids and identified nanoparticles suitable for intratracheal administration through high-throughput screening [230]. The process for high-throughput screening of LNPs involved adding a lipid-ethanol mixture to the aqueous phase in a 96-well plate, followed by thorough mixing of the two phases using a pipette. These validated LNPs efficiently delivered CRISPR-Cas9 mRNA to the mouse airway epithelium for gene editing. This approach not only promises effective treatment for congenital lung diseases but also emphasizes the potential of inhalation delivery in therapeutic gene editing.

**Fig. 13:**
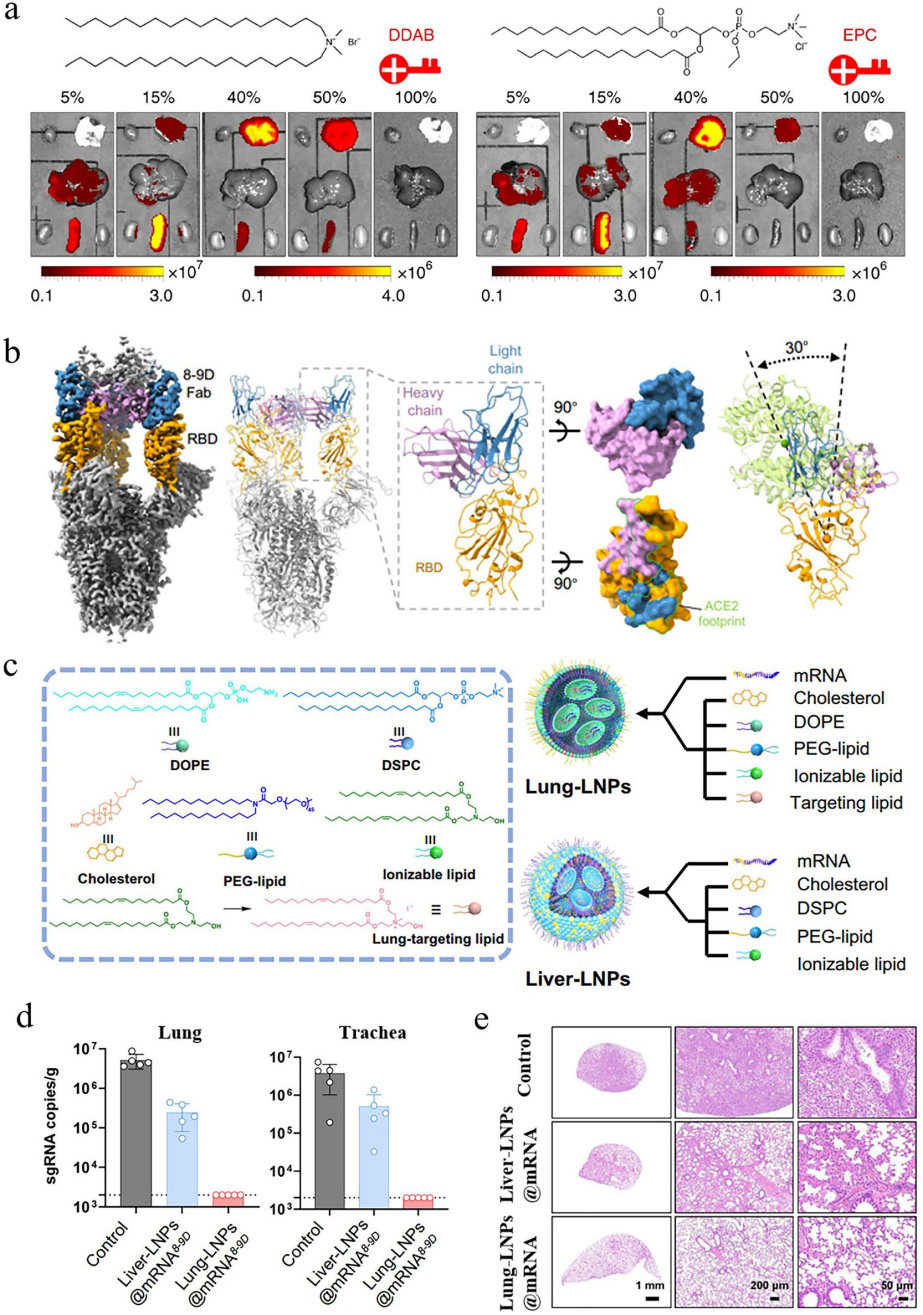
Permanent cationic lipids added to LNPs for lung delivery of mRNA. **a** Cationic lipid DDAB or EPC was added to traditional four-component LNPs, which achieved lung-targeted mRNA delivery. Reproduced with permission from Ref. [20]. Copyright 2020, Springer Nature. **b** Three-dimensional structure of the 8–9D protein. The “up” receptor-binding domains are shown in orange, the heavy chain of 8–9D is in pink, and the light chain is in blue. **c** Schematic diagram of the preparation of lung-targeted LNPs using a five-component delivery system and mRNA. **d** Compared to the control group, the lung-LNPs@mRNA^8–9D^ group showed nearly 100% protection efficiency against SARS-CoV-2 variants. **e** Histopathological results indicated that treatment with lung-LNPs@mRNA^8–9D^ did not cause lung damage. (**b–e**) Reproduced with permission from Ref. [231]. Copyright 2023, Springer Nature

In addition to inhalation delivery, intravenous administration can also deliver mRNA to the lungs, but it requires improvements to traditional delivery systems to avoid uptake or clearance by the liver. In a groundbreaking study, Cheng and his team developed a strategy called selective organ targeting (SORT), which ensured precise mRNA delivery to specific organs (lungs, spleen, or liver) by incorporating an additional component into conventional LNPs [20]. As shown in Fig. 13a, experimental results demonstrated that the incorporation of permanent cationic lipids such as 1,2-dimyristoyl-sn-glycero-3-ethylphosphocholine (EPC), DDAB, or DOTAP to LNP components significantly enabled lung-specific drug delivery. Further investigation into endogenous targeting mechanisms indicated that this lung-specific targeting was largely influenced by changes in the types and quantities of proteins constituting the protein corona in the blood [22]. Tai et al. identified a critical human monoclonal antibody named 8-9D, which exhibited extensive neutralization efficacy against the SARS-CoV-2 variants (Fig. 13b) [231]. Subsequently, they developed the lung-targeting LNPs (lung-LNPs) by adding an additional cationic lipid to the traditional four-component liver-targeting LNPs (Fig. 13c). Impressively, 8-9D mRNA encapsulated in lung-LNPs (lung-LNPs@mRNA^8–9D^) effectively expressed neutralizing antibodies, which prevented the invasion of SARS-CoV-2 variants with good biosafety (Fig. 13d, e). However, five-component mRNA formulations may face stability challenges. To address this issue, a study replaced ionizable lipids in the five components with the polymer PBAE, which also achieved lung delivery of mRNA [232]. By increasing the binding strength of aliphatic chains and charge repulsion between nanoparticles, the stability of the five-element nanoparticles was significantly improved. It was demonstrated that the lyophilized five-element nanoformulations can be stored at 4 °C for at least 6 months, indicating their considerable promise for treating lung diseases.

Developing novel delivery systems and modifying carriers with ligands are two important approaches for mRNA lung delivery. As a typical example, a study introduced amide bonds into ionizable lipids via Michael addition reactions, and the assembled nanoformulations were effectively delivered to the lungs [233]. Researchers observed that the surface of such carriers contained unique protein coronas compared to liver-targeted LNPs, which may be the main reason for lung targeting. In a preclinical model, these amide bond-mediated LNPs showed tremendous potential in treating lymphangioleiomyomatosis, a lung disease caused by abnormal expression of the tuberous sclerosis complex 2 gene. Besides, novel ionizable lipids containing Si bonds promoted mRNA transport to the lungs, further improving lung vascular repair after viral infections [234]. Modifying ligands on the surface of carriers is also an important targeting strategy, as the affinity of ligands can effectively regulate the distribution of carriers in target cells. Plasmalemma vesicle-associated protein (PV1) is a recognized caveolae-associated protein, which constitutes an essential part of the total endothelial membrane of lung capillaries. According to reports, Li et al. developed PV1-modified LNPs by coupling maleimide and cyclopentadiene non-natural amino acids [235]. The subsequent study indicated that PV1-LNPs served as an effective platform for delivering mRNA to the lungs, contributing to the management of diverse respiratory conditions like cystic fibrosis and lung cancer. A similar study coupled platelet endothelial cell adhesion molecule-1 antibodies with LNPs, resulting in nearly a 200-fold increase in mRNA delivery efficiency to the lungs [172]. Therefore, a deep understanding of the physiological characteristics of the lungs and drug delivery systems will promote the development of related fields, bringing more hope and opportunities for human health.

### Spleen Targeting

The spleen is an important immune organ in the human body, capable of removing aged red blood cells and pathogenic microorganisms and promoting antibody production [236, 237]. In recent years, mRNA therapy targeting the spleen has gradually garnered attention. This treatment method involves introducing specific genes to repair genetic defects or protein loss within the spleen, thereby strengthening the immune response or treating diseases related to the spleen. Targeted mRNA therapy for the spleen offers prospects for the management of genetic diseases, infectious diseases, autoimmune diseases, and cancer, with the potential to reduce treatment costs and minimize adverse reactions. Reportedly, mRNA nanomedicines are usually transported to the liver and spleen after intravenous administration [238, 239]. Despite targeted delivery to the spleen seeming straightforward, the reality is that over 80% of mRNA nanomedicines are ultimately taken up by the liver, indicating the challenges that still need to be addressed [240].

**Fig. 14:**
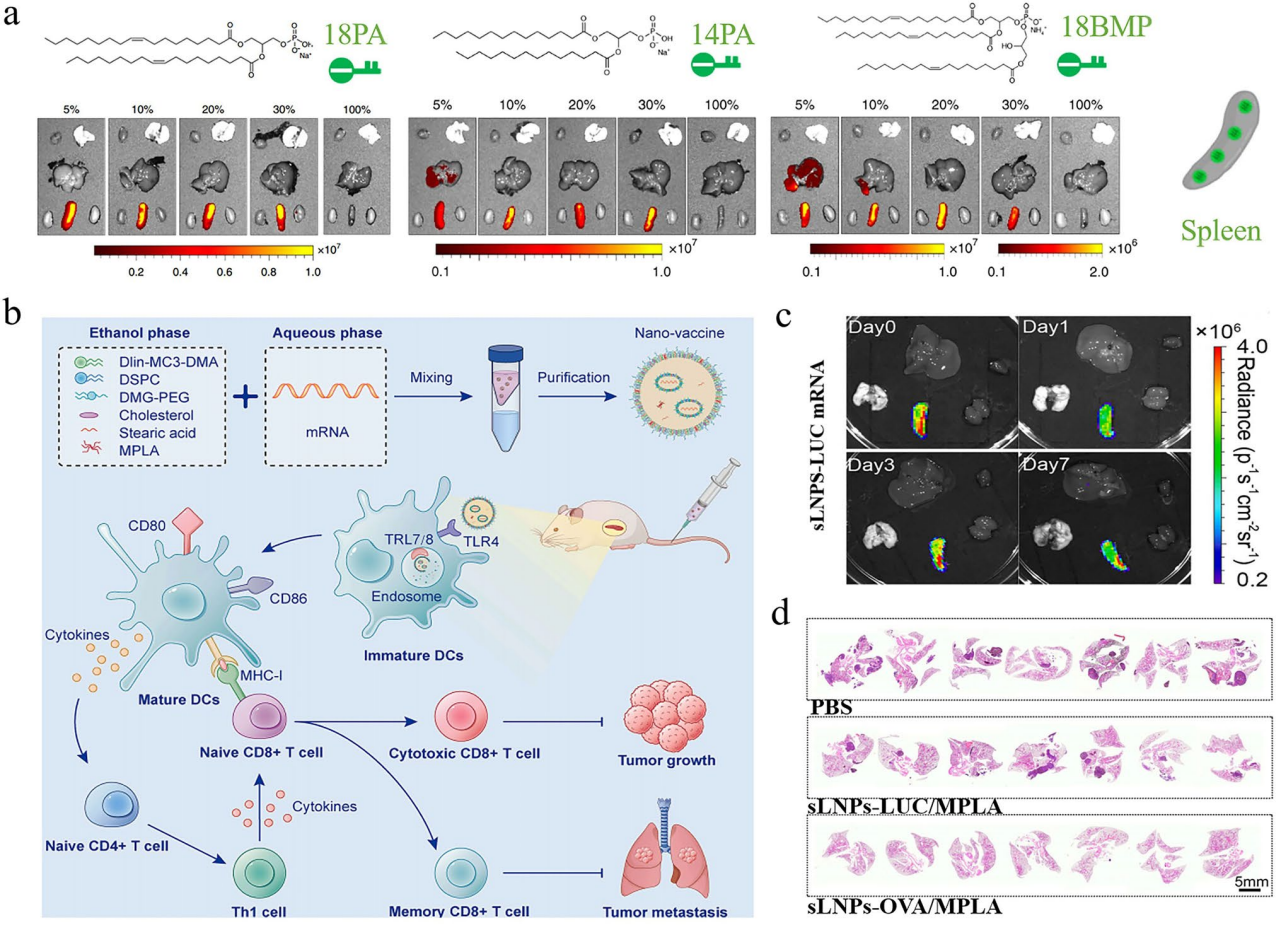
Five-component lipid systems are used for spleen-targeted mRNA delivery. **a** In the conventional four-component system, the addition of anionic lipids such as 18PA, 14PA, and 18BMP enabled mRNA delivery to the spleen. Reproduced with permission from Ref. [20]. Copyright 2020, Springer Nature. **b** An enhanced mRNA vaccine composed of a five-component delivery system and a TLR4 agonist for cancer immunotherapy. **c** After seven days of storage, the five-component delivery system still had the spleen delivery function. **d** After receiving different treatments, the HE staining of mouse tumors showed that sLNPS-OVA/MPLA formulations can effectively inhibit melanoma growth. (**b–d**) Reproduced with permission from Ref. [241]. Copyright 2023, Elsevier

Unlike the lungs, which feature a substantial surface area and distinct physiological functions, targeting the spleen through changes in the method of administration is challenging. For lipid materials, common strategies for spleen-targeted delivery include adjusting formulations, developing new ionizable lipids, or combining both approaches [240]. As mentioned earlier, adding a fifth cationic lipid to traditional four-component lipids enhanced mRNA delivery to the lungs. In contrast, incorporating permanent anionic lipids such as 1,2-dioleoyl-sn-glycero-3-phosphate (18PA), 1,2-dimyristoyl-snglycero-3-phosphate (14PA), and sn-(3-oleoyl-2-hydroxy)-glycerol-1-phospho-sn-3'-(1',2'-dioleoyl)-glycerol (18BMP) significantly improved mRNA delivery to the spleen (Fig. 14a) [20]. Mechanistically, this liver-bypassing spleen targeting resulted from the distinct proteins adsorbed on the lipid surface. As shown in Fig. 14b, a similar study introduced additional stearic acid into four-component LNPs, enhancing their structural stability and delivery potential [241]. As expected, the five-component delivery system successfully delivered OVA mRNA to the spleen (Fig. 14c). By adding TLR4 agonists, the resulting mRNA vaccines triggered a series of immune responses and halted the growth of EG.7-OVA tumors through sustained memory protection (Fig. 14d).

**Fig. 15:**
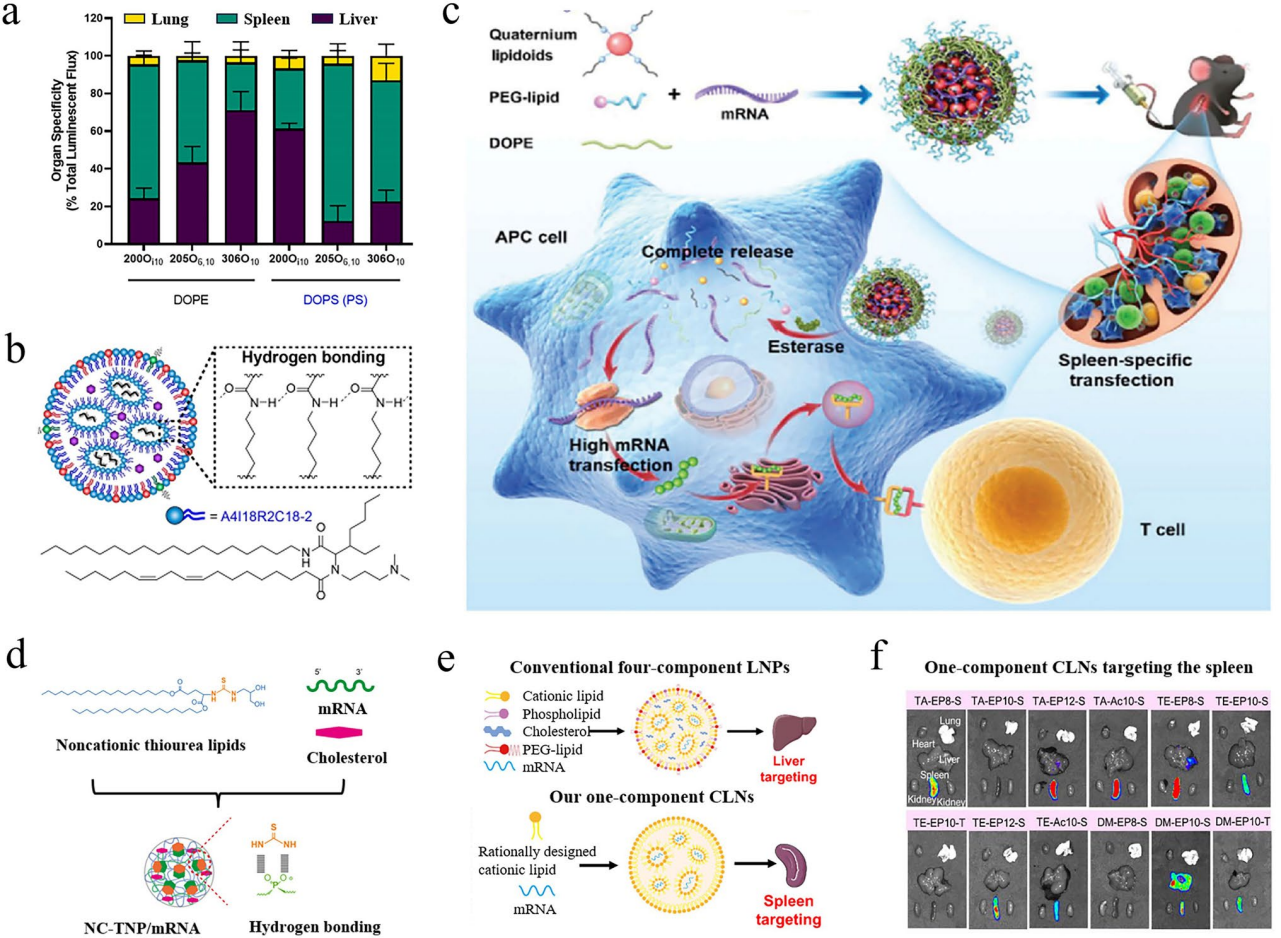
Four-component, three-component, two-component, and one-component lipid systems are employed for spleen-targeted delivery. **a** After replacing the neutral lipids in LNPs with net negatively charged lipids, the data showed increased mRNA expression in the spleen. Reproduced with permission from Ref. [242]. Copyright 2022, Elsevier. **b** An ionizable lipid containing double amide bonds was developed, and its derived LNPs were capable of delivering mRNA to the spleen. Reproduced with permission from Ref. [243]. Copyright 2023, Wiley − VCH. **c** Schematic illustration of mRNA spleen delivery using the three-component carrier. Reproduced with permission from Ref. [244]. Copyright 2023, Wiley − VCH. **d** Unlike traditional electrostatic adsorption, the two-component mRNA carrier relies on hydrogen bonding to compress mRNA. Reproduced with permission from Ref. [245]. Copyright 2023, National Academy of Sciences. **e** Schematic diagram compared the selective organ delivery differences between the four-component delivery platform and the one-component delivery platform. **f** Research data showed that one-component carriers with different chemical structures affected spleen delivery efficiency. (**e, f**) Reproduced with permission from Ref. [246]. Copyright 2024, Wiley − VCH

Although five-component delivery systems are highly effective for achieving precise delivery, some studies suggest that this drug delivery strategy further complicates an already complex formulation, potentially slowing down the drug review process. To optimize delivery systems, four-component, three-component, two-component, and even single-component carriers have also garnered significant interest from scientists, particularly in studies related to spleen delivery. One study explored strategies to maintain the four-component nanoparticle system while achieving targeted transformation [242]. Researchers substituted the standard helper lipid DOPE with anionic lipids like phosphatidic acid, phosphatidylglycerol, and phosphatidylserine, resulting in mRNA expression predominantly in the spleen (Fig. 15a). This replacement altered the liver-to-spleen protein expression ratio from 8:1 to 1:3. He et al. reported a strategy for constructing a large library of ionizable lipids using the Ugi four-component reaction [243]. This one-pot method could occur under mild conditions, avoiding lengthy reaction and purification processes. After screening and validation, a novel ionizable lipid A4I18R218-2 with double amide bonds, can form hydrogen bonds within LNPs, thereby enhancing their stability (Fig. 15b). More importantly, by generating ionizable lipids with new skeletons, the prepared four-component nanoformulations exhibited spleen-targeting capabilities. As shown in Fig. 15c, another key study developed esterase-triggered quaternium lipid-like molecules, which demonstrated the targeting potential of the three-component delivery system [244]. Typically, the lipidoid molecules carry a positive charge under normal physiological conditions, but their charge reverses upon encountering esterase, quickly gaining a negative charge. The three-component lipids, including cationic lipids, PEG lipids, and DOPE, were assembled into nanoparticles with OVA mRNA at a molar ratio of 78.5:1.5:20. These nanoparticles can selectively express in the spleen and induce a strong antigen-specific immune response. Additionally, some studies have developed two-component and even single-component delivery systems for spleen targeting, aiming to simplify the preparation of mRNA nanomedicines and enhance the specificity and efficacy of therapeutic interventions (Fig. 15d-f) [245, 246].

Careful design of polymer carriers also has the potential for spleen delivery. A study conducted by McKinlay et al. developed a new library based on amphiphilic charge-altering releasable transporters (CARTs), successfully transfecting lymphocytes both in vitro and in vivo [247]. Through lipid diversification, certain binary mixtures increased mRNA transfection efficiency from 9%–12% to 80%. Further research at the cellular level confirmed that these optimized CART mixtures effectively transfected lymphocytes, particularly B and T cells in the spleens. Biodegradable polymer carriers have significant application value in spleen-targeted delivery. It is well known that biomaterials containing disulfide bonds can be degraded in a reducing environment, and this property is usually utilized to design smart drug delivery systems. For instance, biodegradable poly(beta-amino ester) nanocarriers based on disulfide bonds were developed for cancer vaccines [248]. Of note, these nanocarriers demonstrated a strong ability to target dendritic cells (DCs) in the spleen without the need for surface functionalization. By co-delivering with TLR agonists, the polymer carriers enhanced antigen-specific CD8^+^ T cell responses, effectively inhibiting tumor growth in various mouse tumor models. Besides LNPs and polymer carriers, other delivery systems such as amphiphilic carbon dots and cell-penetrating peptide NF424 have also shown promising results, offering more options for achieving spleen-targeted delivery [249, 250].

### Heart Targeting

The heart is the driving organ of the circulatory system, responsible for pumping blood to deliver oxygen and nutrients to tissues and organs throughout the body [251]. However, cardiovascular diseases pose significant health challenges globally, including conditions such as myocardial infarction, arrhythmias, angina, and heart failure. These diseases can be caused by various elements, including genetics, lifestyle choices, and environmental factors. mRNA technology harnesses the body's molecular mechanisms to guide cells to produce specific proteins, offering promising avenues for treating cardiovascular diseases. Specifically, mRNA therapy facilitates heart repair and regeneration by supplying damaged myocardial cells with the necessary proteins. Common delivery strategies include intramyocardial injection, development of targeted nanocarriers, or combination with stem cell therapy, all of which have the potential to manage heart-related diseases.

**Fig. 16:**
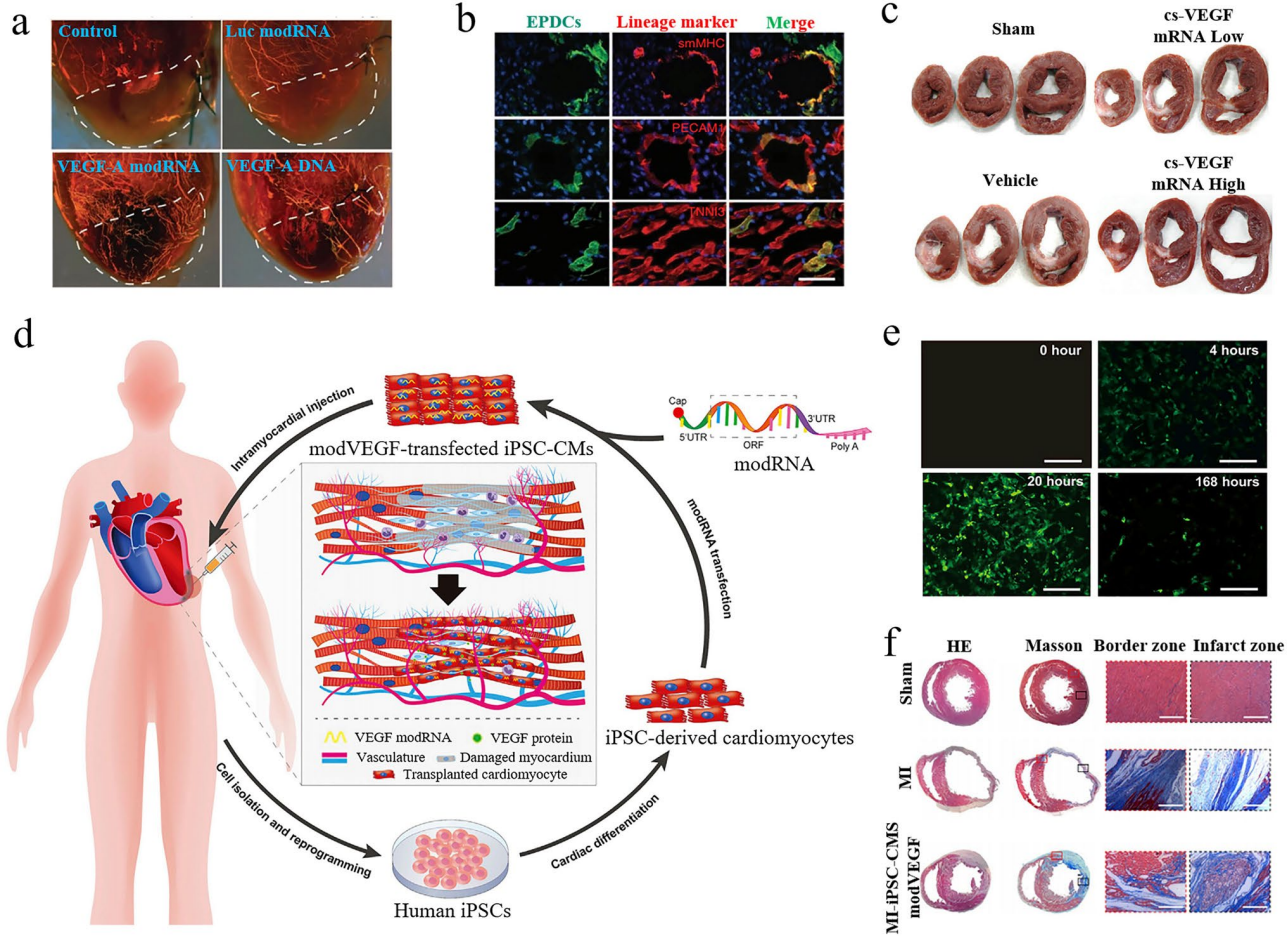
Heart targeting of mRNA achieved through intramyocardial injection. **a** After VEGF mRNA administration to the myocardium, vascular density significantly increased in the infarcted region. **b** Immunofluorescence images indicated that VEGF mRNA effectively promoted the mobilization and differentiation of epicardial progenitor cells. (**a, b**) Reproduced with permission from Ref. [256]. Copyright 2013, Springer Nature. **c** A study used VEGF mRNA dissolved in a citrate saline buffer to treat myocardial infarction. Two months after treatment, the hearts of the experimental mice were collected and sliced. Reproduced with permission from Ref. [257]. Copyright 2018, Cell Press. **d** Illustration of cell therapy combined with mRNA technology for treating heart-related disease. **e** After transfecting iPSC-CMs with eGFP mRNA for 168 h, a small amount of green fluorescence was still detected. **f** Four weeks after treatment with iPSC-CMs transfected with VEGF mRNA, the rat hearts were collected and analyzed by staining. (**d, f**) Reproduced with permission from Ref. [258]. Copyright 2023, Cell Press

Intramyocardial injection is a promising drug delivery route, as it allows drugs to reach myocardial tissue more quickly while avoiding the uptake of mRNA nanomedicines by the liver. Vascular endothelial growth factor (VEGF) is a key member of the growth factor family. It mainly stimulates cell proliferation, migration, and angiogenesis and has been suggested as a therapeutic agent to enhance myocardial function [252–255]. Through intramyocardial injection, modified VEGF mRNA significantly improved vascular density and cardiac function in the infarcted area, while other experimental groups did not meet the expected outcomes (Fig. 16a) [256]. This was attributed to VEGF mRNA accelerating migration and directional differentiation of epicardial progenitor cells (Fig. 16b). It should be noted that this work employed naked mRNA without relying on any functional carriers. A similar study reported that VEGF mRNA dissolved in a citrate-saline buffer was injected into the heart, resulting in sustained expression of the expected protein and reduced area of myocardial fibrosis (Fig. 16c) [257]. These results were also validated in both small and large animal models of permanent occlusive myocardial infarction. Given the persistence and reparative function of cell therapy, combining mRNA technology with cell therapy holds promise for reprogramming cells and aiding in the management of heart-related diseases. As shown in Fig. 16d, Ai and his colleagues combined chemically modified VEGF mRNA and induced pluripotent stem cell-derived cardiomyocytes (iPSC-CMs) to investigate the efficacy of myocardial repair [258]. To visualize protein expression, enhanced green fluorescent protein (eGFP) mRNA was selected for transfection of iPSC-CMs. After transfection for 168 h, strong green fluorescence was still observed, indicating sustained expression of the target protein within the cell (Fig. 16e). Importantly, animal experiments confirmed that these engineered iPSC-CMs enhanced cellular growth, mitigated oxygen deprivation in the infarcted region, and resulted in the recovery of cardiac function (Fig. 16f).

**Fig. 17:**
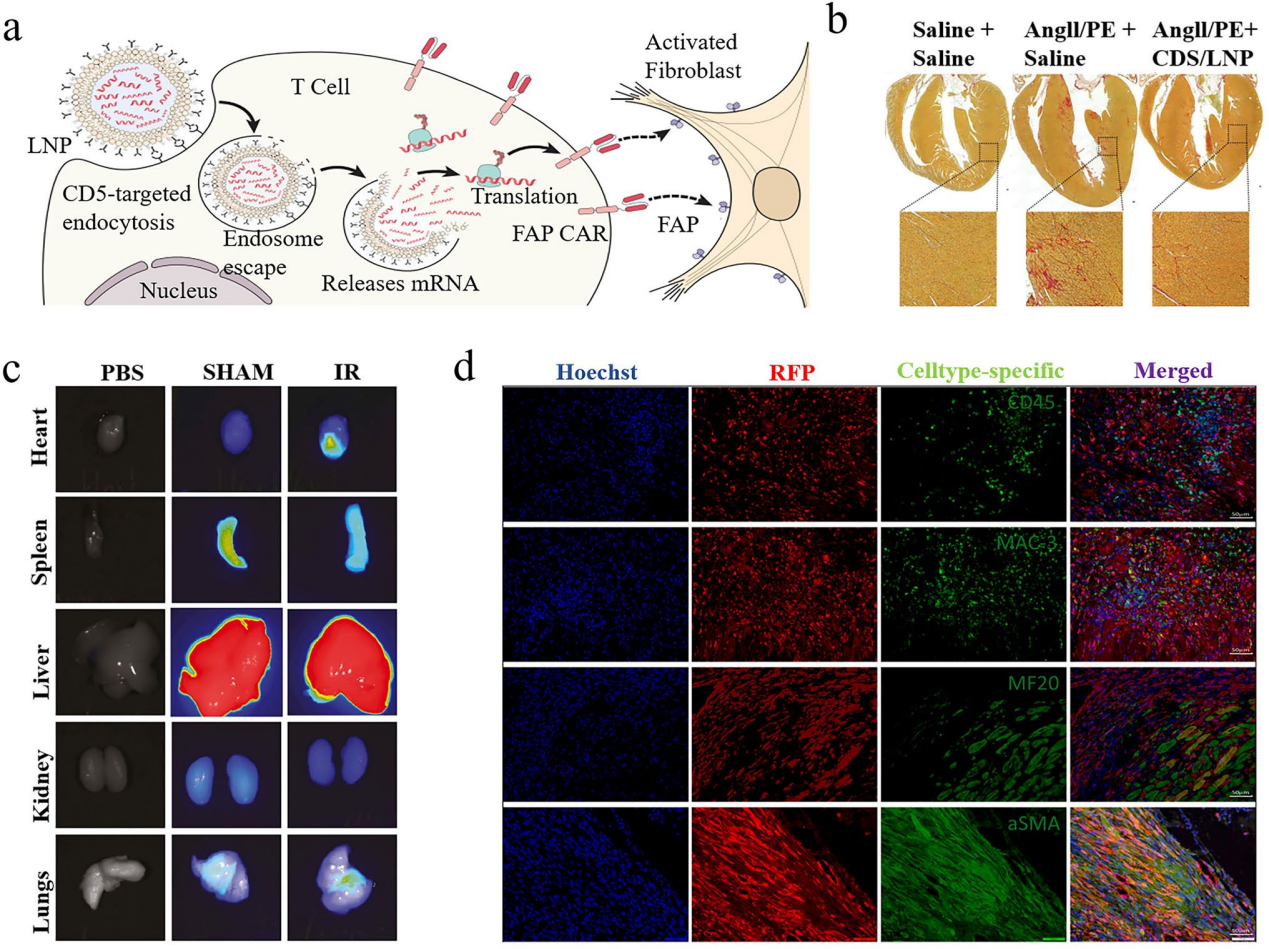
Other strategies for heart-targeted delivery. **a** Schematic diagram of CD5-targeted LNPs encapsulating CAR mRNA for the treatment of cardiac injury. **b** Picrosirius red staining of the myocardium showed a significant enhancement in mouse cardiac function after receiving CD5/LNP treatment. (**a**, **b**) Reproduced with permission from Ref. [259]. Copyright 2022, American Association for the Advancement of Science. **c** Through intravenous administration, it was observed that small amounts of mRNA nanomedicines could reach the heart smoothly. **d** Further analysis demonstrated that LNPs were mainly taken up by cardiac fibroblasts in the heart. (**c**, **d**) Reproduced with permission from Ref. [262]. Copyright 2022, Elsevier

Despite the high specificity of direct intramyocardial injection, there are challenges and limitations in its clinical applications. Firstly, direct injection requires specialized medical equipment and techniques, thereby increasing complexity and cost. Secondly, the unique structure of myocardial tissue and the dynamic movement of the heart may affect the accuracy and efficacy of injections, potentially leading to variability in treatment outcomes. Additionally, direct injection may carry risks of local inflammation or tissue damage, particularly with repeated injections. To overcome these challenges, researchers are exploring alternative approaches and technologies for treating cardiovascular diseases. One promising strategy involves using targeted nanocarriers to deliver mRNA directly to cardiac tissue via intravenous administration. For instance, a groundbreaking study utilized CD5-targeted LNPs to encapsulate mRNA encoding CAR (Fig. 17a) [259]. First, the thiolated anti-human CD5 was reacted with DSPE-PEG-Mal to obtain the DSPE-PEG-CD5 lipid, which was then assembled with other lipids and mRNA to form CD5-targeted LNPs. Following intravenous administration, these mRNA nanomedicines can reprogram T cells and selectively attack activated fibroblasts. Analysis on a mouse model revealed that this innovative therapeutic approach effectively reduced fibrotic area and restored cardiac function (Fig. 17b). The advancement of this technique lies not only in imparting specific targeting capabilities to LNPs but also in leveraging mRNA technology to generate effective CAR T cells in vivo. Interestingly, some studies report that modified mRNA can also be delivered to the heart by systemic administration without any treatment of LNPs, which may be related to the type of disease. For example, coronary artery occlusion causes vascular endothelial leakage, allowing nanomedicines to extravasate from the circulation in the ischemic regions [260, 261]. According to research by Evers et al., some LNP formulations were expressed in the mouse heart after intravenous injection (Fig. 17c) [262]. Further fluorescence staining at the cellular level showed that LNP formulations were mainly taken up by cardiac fibroblasts, with a portion also being engulfed by macrophages or cardiomyocytes (Fig. 17d). Although the expression levels of LNP formulations in the heart are lower compared to the liver, there still exists potential for treating cardiac diseases.

### Brain Targeting

As the control center of the human body, the brain regulates a myriad of functions including sensation, movement, emotion, and cognition, and boasts a complex neural network [263–265]. When the brain's function is impaired or neural networks encounter issues, it can lead to various brain disorders. These disorders encompass neurodegenerative diseases, brain tumors, strokes, and epilepsy, which are typically treated through medication, surgery, and rehabilitation therapies [266–268]. mRNA therapy, as an emerging biomedical technology, has shown promising results in treating brain disorders. Although brain injection is a direct targeted delivery method, it poses significant challenges, not only requiring high-level technical expertise and equipment support but also holding extremely high risks. Thus, scientists are engineering various carrier systems to promote mRNA through the BBB via systemic administration, further reducing risks and enhancing therapeutic efficacy [269–271].

**Fig. 18:**
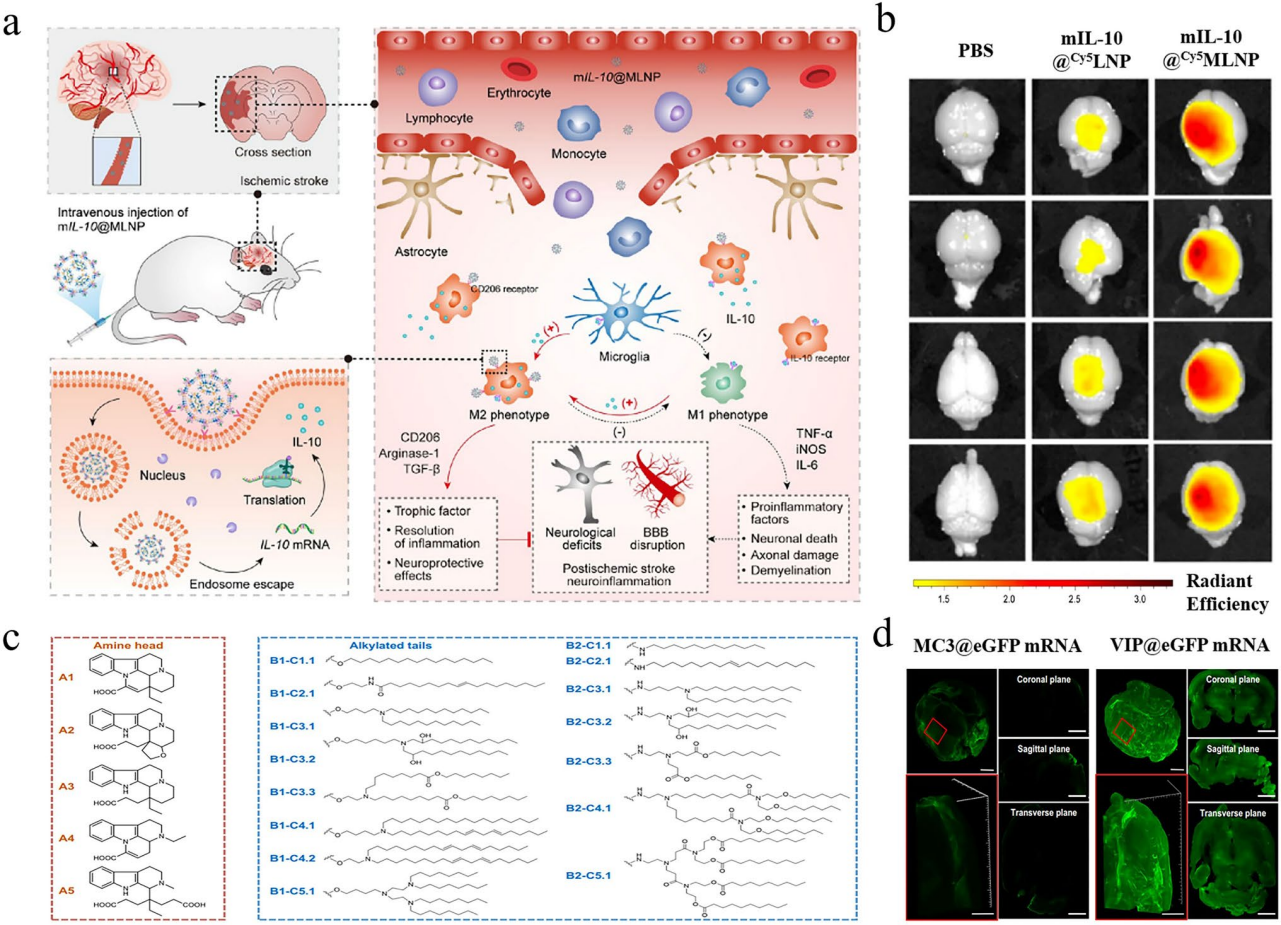
Modified lipid materials for mRNA brain delivery. **a** Schematic diagram of MLNP encapsulating IL-10 mRNA for the treatment of ischemic stroke. **b** Compared to LNPs, the MLNP delivery systems allowed more mRNA to accumulate in the brain. (**a, b**) Reproduced with permission from Ref. [272]. Copyright 2024, American Chemical Society. **c** Chemical structure of ionizable lipids based on vinpocetine. **d** Compared to MC3 lipids, the vinpocetine-derived LNPs efficiently delivered eGFP mRNA to the mouse brain. (**c, d**) Reproduced with permission from Ref. [275]. Copyright 2024, Springer Nature

Recently, a study developed mannose-modified LNPs (MLNP) that encapsulate interleukin-10 (IL-10) mRNA and target M2-polarized microglia in the ischemic brain region (Fig. 18a) [272]. As shown in Fig. 18b, this nanoformulation, measuring around 90 nm, was capable of crossing the BBB and accessing the brain parenchyma. The expressed IL-10 drove M2 polarization of microglial cells, elevated levels of trophic factors, and restored functionality of damaged brain tissue. Vinpocetine, a compound extracted from Vinca minor leaves, has been suggested as a therapeutic agent for brain-related disorders [273, 274]. As shown in Fig. 18c, Bian et al. utilized the chemical structure derived from vinpocetine as the main scaffold and developed a series of novel ionizable lipids [275]. Compared to the FDA-approved ionizable lipids MC3, the vinpocetine-based delivery systems efficiently delivered eGFP into the brains of model mice, as evidenced by stronger green fluorescence observed in vivo (Fig. 18d). Further investigation confirmed the significant therapeutic potential of these nanoparticles for treating brain diseases, possibly attributed to the retained intrinsic pharmacological activity of vinpocetine. According to reports, carriers modified with ligands such as P-selectin, insulin receptor, transferrin receptor, intracellular adhesion molecule-1 and VCAM-1, possessed the ability to target brain tissues [276–278]. In particular, binding with the VCAM-1 ligand significantly enhanced the accumulation level of nanocarriers in the brain. A study utilized VCAM-modified LNPs for cargo delivery and discovered de novo expression of the loaded mRNA in cell experiments [29]. Following the tail vein injection, the mRNA formulations were successfully transported to the inflamed brain region, which alleviated TNF-α-induced cerebral edema. Taken together, these delivery methods mainly involve targeted modification of lipids, enabling them to cross the BBB through intravenous administration and achieve therapeutic outcomes. It is worth mentioning that microbubble-assisted focused ultrasound (FUS) is also an emerging strategy to facilitate the brain transport of LNPs [279]. With the assistance of FUS, the BBB can be effectively opened, allowing LNP-encapsulated mRNA to successfully reach the brain. The uptake analysis of nanoparticles indicated that exogenous proteins were primarily expressed in microglial cells and CD31-positive endothelial cells, with no detectable expression in astrocytes or neurons.

**Fig. 19:**
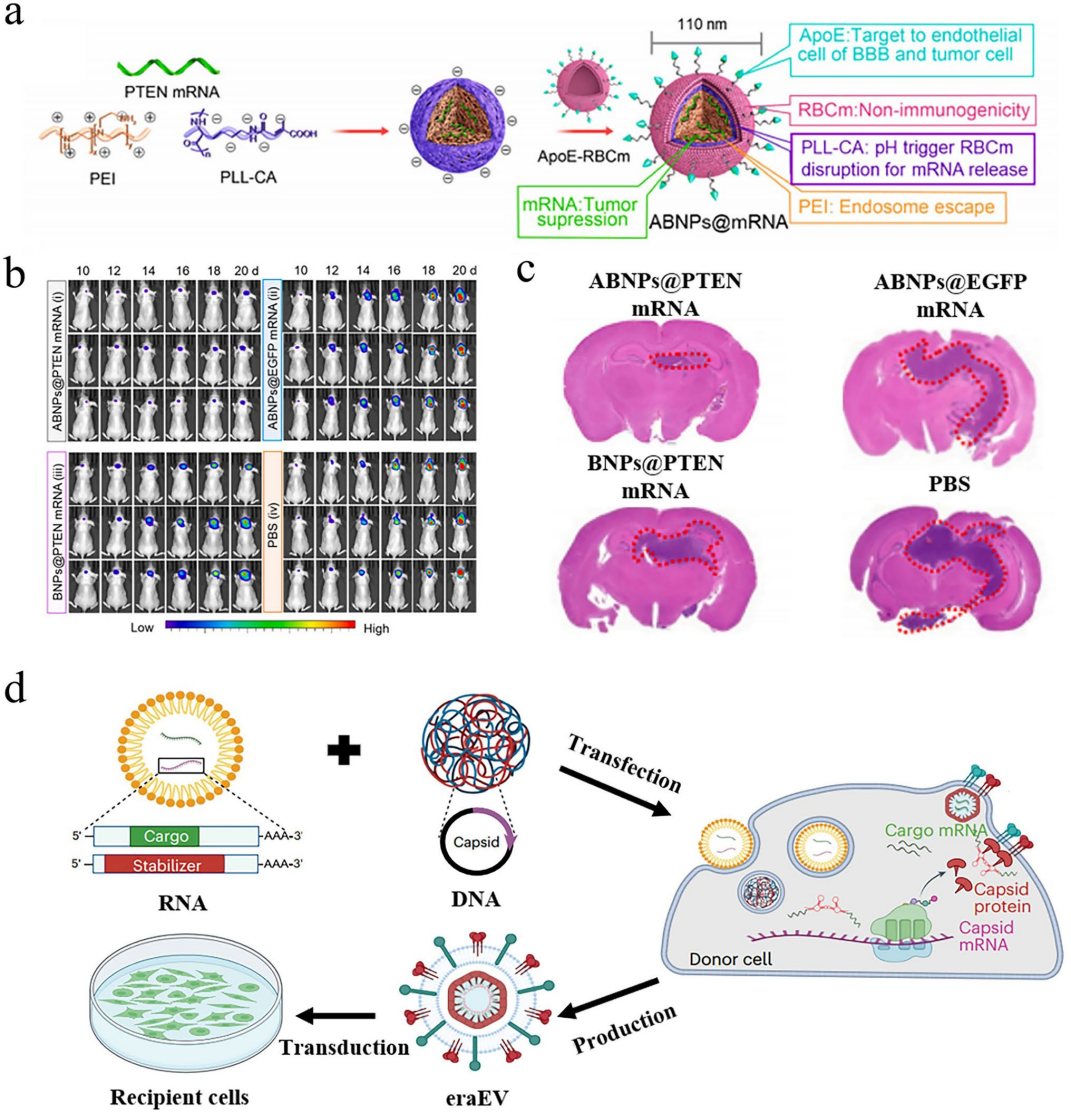
Engineering polymer materials and exosomes for mRNA brain delivery. **a** Preparation of a multi-component polymer carrier with brain-targeting functionality. **b** Compared with other experimental groups, ABNPs encapsulated with PTEN mRNA (ABNPs@PTEN) demonstrated a more robust ability to combat glioblastoma. **c** After receiving ABNPs@PTEN treatment, HE staining indicated a notable enhancement in the brain tumor of the mouse. (**a-c**) Reproduced with permission from Ref. [280]. Copyright 2023, Elsevier. **d** Schematic diagram showed the preparation of exosomes with retrovirus-like protein capsids. Reproduced with permission from Ref. [284]. Copyright 2024, Springer Nature

In addition to lipid materials, engineered polymer materials and extracellular vesicles also contribute to mRNA delivery to the brain. As shown in Fig. 19a, novel ternary nanoparticles (ABNPs) composed of PEI, citraconic anhydride-grafted poly-L-lysine, and ApoE peptide-decorated red blood cell membrane (ApoE-RBCm) achieved mRNA delivery in brain tissue [280]. Since the delivery systems were composed of multiple components, they exhibited multifunctionality, including lysosome escape, pH responsiveness, non-immunogenicity, and targeting of endothelial cells in the BBB. In a mouse model with tumor burden, administration of ABNPs@mRNA formulations significantly inhibited tumor growth compared to other experimental groups, with negligible toxic side effects on major organs (Fig. 19b). HE staining from brain tissue also confirmed this result, underscoring the therapeutic potential of the delivery system (Fig. 19c). Another study compared the delivery efficiency of carriers such as PEI, PEI2k conjugated with dexamethasone (Dexa-PEI2k), and PEI2k conjugated with deoxycholic acid (DA-PEI2k) in the brain [281]. In both cell and animal experiments, polymer DA-PEI2k exhibited the highest delivery efficiency and cell viability, thus aiding in reducing the infarct area and treating ischemic stroke. Interestingly, there's no need for any engineering design of polymer carriers. Simply bundling RNA can also increase gene expression levels in the brain [282]. By using RNA nanotechnology to form bundled structures of mRNA, the stability of polymer micelles in the bloodstream was significantly enhanced, leading to effective protein expression in the brain. For exosome carriers, long mRNA is difficult to load into exosomes and be taken up by neurons [283]. To address this challenge, an innovative technique involved introducing lipid-encapsulated mRNA and polymer-encapsulated DNA into donor cells, generating exosomes with retrovirus-like protein capsids that can effectively load and deliver mRNA (Fig. 19d) [284]. In addition to having stronger cargo loading capacity, engineered exosomes derived from autologous leukocytes exhibited high stability while improving the rate of crossing the BBB.

### Bone Targeting

Bones are a crucial component of the human body, as they support the entire body structure and provide motor function. Besides, bones are also significant in storing minerals, participating in metabolism, and producing blood. However, they are susceptible to various diseases, including fractures, osteoporosis, and bone tumors. In recent years, mRNA technology has gained attention in the medical field, including for the targeted treatment of the skeletal system. By directing the synthetic mRNA to bone tissues, it is possible to stimulate bone cell proliferation and differentiation, which accelerates the healing of bone-related diseases. Bone-targeted mRNA delivery strategies are similar to the methods mentioned earlier, including local administration, combined stem cell therapy, ligand modification of LNPs, etc. These delivery strategies aim to enhance the targeting and biological stability of mRNA in bone tissues, promoting the development of precise treatments for bone-related diseases.

**Fig. 20:**
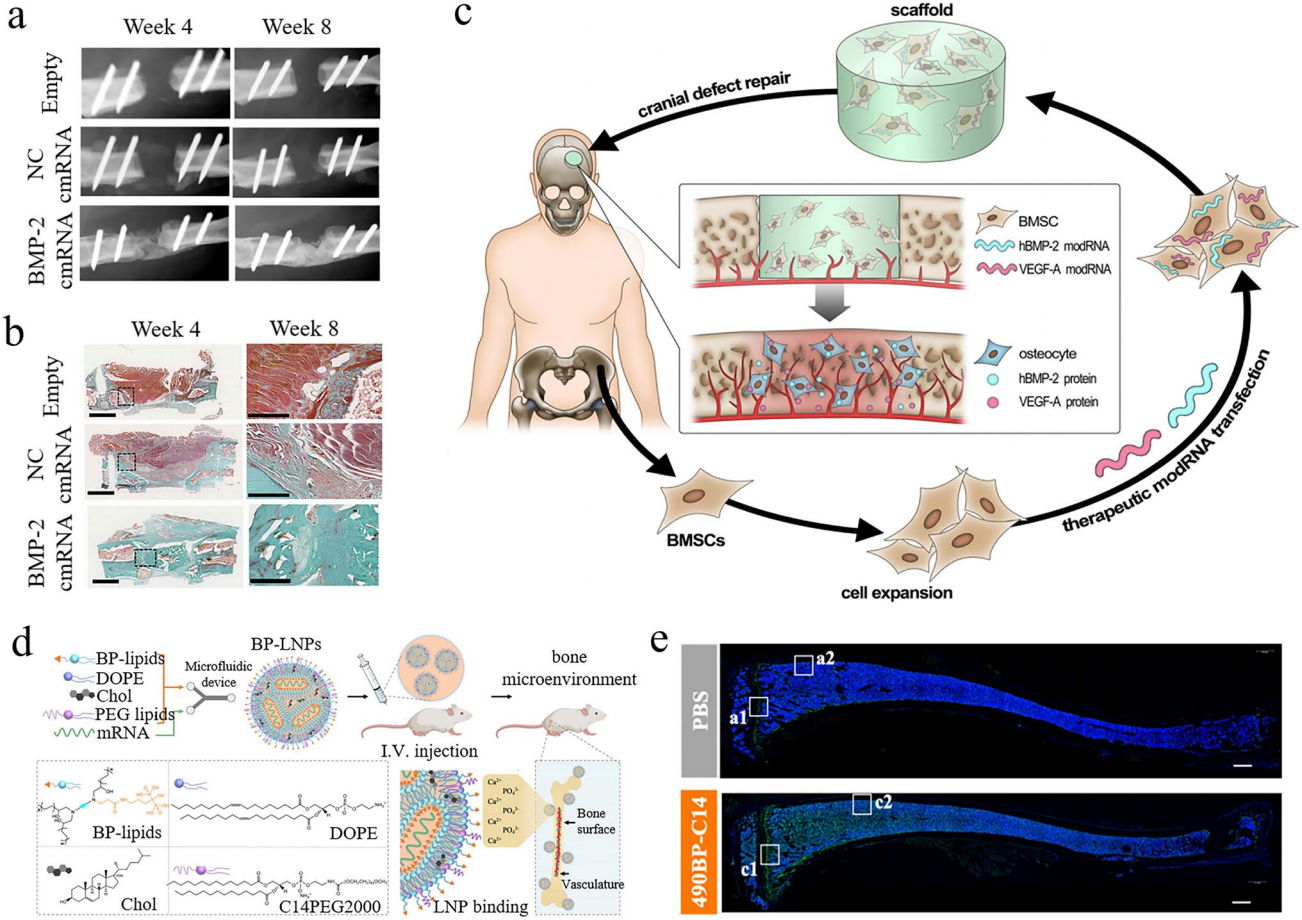
Bone-targeted mRNA delivery strategy. **a** After eight weeks of local administration, chemically modified BMP-2 mRNA promoted the complete healing of the damaged bone. **b** Masson staining indicated that the BMP-2 cmRNA group more effectively induced de novo bone deposition compared to other treatment groups. (**a, b**) Reproduced with permission from Ref. [289]. Copyright 2022, American Association for the Advancement of Science. **c** Schematic diagram of mRNA technology combined with stem cell therapy for repairing cranial bone. Reproduced with permission from Ref. [290]. Copyright 2021, Springer Nature. **d** Illustrative diagram of BP-derived LNPs for bone-targeted delivery. **e** More mRNA formulations were accumulated in the bone compared to the PBS group, as evidenced by the significant green fluorescence observed. (**d, e**) Reproduced with permission from Ref. [294]. Copyright 2022, American Chemical Society

Recombinant human bone morphogenetic protein 2 (rhBMP-2) is a significant bioactive protein, particularly effective in promoting fracture healing, repairing skeletal defects, and facilitating spinal fusion [285, 286]. However, this therapy needs large doses, leading to the occurrence of side effects and an increase in treatment costs [287, 288]. Using mRNA therapy to express rhBMP-2 is a promising candidate solution to address the challenges mentioned above. Through local injection, a study targeted the delivery of mRNA formulations encoding BMP-2 to a segmental defect model [289]. As shown in Fig. 20a, the BMP-2 cmRNA group exhibited optimal bone repair effects after four weeks of treatment, particularly by the eighth week, when the bone defect was nearly completely healed. Compared to the Empty group and NC cmRNA group, Masson staining indicated that the BMP-2 cmRNA group can more effectively induce de novo bone deposition, as evidenced by the observation of a denser green matrix (Fig. 20b). Local administration of mRNA-transfected stem cells is also an effective approach to achieving bone targeting [290]. As depicted in Fig. 20c, stem cells extracted from the iliac bone marrow were expanded and modified with VEGF-A and hBMP-2 mRNA. Subsequently, these cells were seeded onto scaffolds and implanted into the bone defect site in the human body. The research results confirmed the efficacy of combining mRNA technology with stem cell therapy in stimulating bone healing and regeneration, with the additional benefits of safety and cost-effectiveness.

In addition to local injection, intravenous administration is also effective for bone-targeted delivery of mRNA, but it requires engineering modifications to the delivery system. Previous studies have shown that bisphosphonates (BP) have a strong affinity for bones because they are analogs of inorganic pyrophosphate and readily chelate with calcium ions (Ca^2+^) in bones [291–293]. Taking advantage of this property, Xue et al. developed BP-derived LNP delivery systems with bone-targeting capability, as shown in Fig. 20d [294]. Following intravenous administration, the strong interaction between the BP and hydroxyapatite improved the accumulation of LNPs in the bone microenvironment, as more green fluorescence was observed in the experimental group (Fig. 20e). Hematopoietic stem cells (HSCs) usually reside in the bone marrow and are precursor cells responsible for producing blood cells [295]. However, delivering nucleic acid molecules to HSCs in the bone marrow remains challenging, particularly in patients with hematological diseases. CD117 is considered an important marker for HSCs and is key to achieving bone-targeted delivery systems. By engineering modifications to PEG lipids, the resultant anti-CD117-labeled lipid carriers can effectively deliver gene editing systems to the bone marrow and correct HSCs [171]. This technology has significant safety as it does not require the transplantation of HSCs. A recent study added covalent lipids to a four-component-based LNP delivery system and surprisingly found that mRNA was delivered to various cells in the bone marrow, including HSCs, B cells, leukemia cells, and others [296]. Proteomic analysis of serum proteins on the surface of these nanoparticles showed that the bone marrow homing function primarily depended on ApoE.

### Other Targeting Technologies

**Fig. 21:**
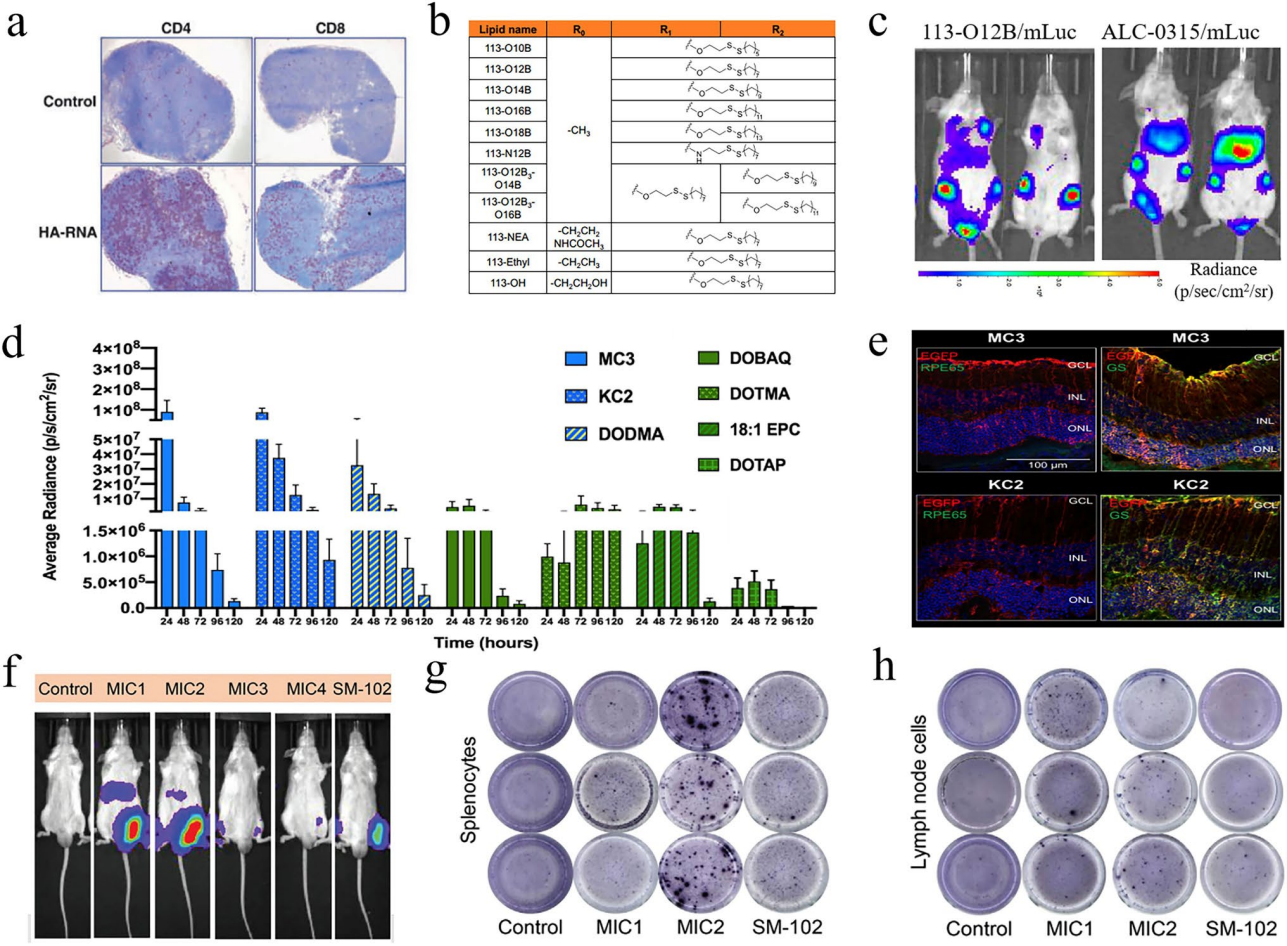
Strategies for targeting other organs or tissues. **a** After intranodal immunization, the quantity of CD4^+^ and CD8^+^ T cells in the experimental group increased rapidly. Reproduced with permission from Ref. [297]. Copyright 2010, American Association for Cancer Research. **b** Ionizable lipids with lymph node-targeting ability. **c** After subcutaneous injection of LNP formulations, it was found that the ionizable lipid 113-O12B derived LNPs exhibited higher lymph node delivery efficiency compared to the ALC-0315 LNPs. (**b, c**) Reproduced with permission from Ref. [298]. Copyright 2022, National Academy of Sciences. **d** Comparison of delivery efficiency of different lipid materials in the eye. **e** Lipids MC3 and KC2 primarily deliver mRNA to the retinal pigmented epithelium, with a smaller amount reaching the Müller glia. (**d, e**) Reproduced with permission from Ref. [299]. Copyright 2019, Elsevier. **f** Using intramuscular injection, a substantial amount of mRNA expression was observed in the muscle, particularly in the MIC1 and MIC2 groups. **g, h** After secondary stimulation, ELISpot assays were used to detect the number of T cells in splenocytes and lymph node cells. (**f, h**) Reproduced with permission from Ref. [313]. Copyright 2022, Wiley − VCH

In addition to the previously mentioned delivery targets, many other functional tissues and organs have also become focuses of mRNA delivery, including lymph nodes, eyes, pancreas, and muscles. For lymph node-targeted mRNA delivery, intranodal administration is a direct and efficient method. Targeting mRNA-based nanomedicines to lymph nodes can effectively induce immune-related responses, offering therapeutic potential in cancer, infectious diseases, and autoimmune disorders. As a typical case, Kreiter et al. injected naked mRNA encoding influenza virus hemagglutinin-A directly into the lymph nodes [297]. On the fifth day after administration, frozen sections of the lymph nodes showed a rapid expansion of CD4^+^ and CD8^+^ T cells in the experimental group, whereas this was absent in the control group (Fig. 21a). Further experiments confirmed that intranodal administration generated more sustained immune responses compared to intradermal and subcutaneous administration. As shown in Fig. 21b, c, a study developed a series of novel ionizable lipids, among which 113-O12B-derived LNPs demonstrated the highest lymph node delivery efficiency, even surpassing that of ALC-0315 lipid-derived LNPs [298]. Notably, these lymph node-targeting mRNA formulations, administered via subcutaneous injection, exhibited significant anti-tumor effects in the B16F10 melanoma model.

Ocular therapy using mRNA technology has broad prospects due to its relative immune privilege, target accessibility, and low therapeutic dosages. By directly injecting mRNA drugs, there is potential to cure ocular diseases such as retinal disorders, corneal injuries, glaucoma, and macular degeneration. In a foundational study, seven LNP variants were tested to investigate their effectiveness in delivering mRNA to the back of the eye [299]. As shown in Fig. 21d, the ocular delivery efficiency of the three ionizable lipid-derived LNPs was significantly higher than that of the cationic lipid-derived LNPs, with the lipid materials MC3 and KC2 being particularly notable. The primary site of mRNA expression delivered by MC3- and KC2-based LNPs was the retinal pigmented epithelium, and a small portion of the expression also occurred in Müller glia. (Fig. 21e). Another strategy involved combining modified mRNA with adipose-derived mesenchymal stem cells (ADSCs) for corneal repair [300]. After receiving subconjunctival injection of mRNA-engineered ADSCs, the model mice showed corneal nerve regeneration and restoration of corneal function, demonstrating the effectiveness of this approach for treating ocular diseases. For pancreatic delivery, Melamed et al. formulated three types of LNPs, each containing unique ionizable lipids: 306O_i10_, 200O_i10_, or 514O_6,10_ [24]. Through intraperitoneal administration, these LNPs can generate strong and specific protein expression in the pancreas. This discovery makes it possible to cure diabetes and cancer related to pancreas.

Muscle is an important tissue involved in various physiological activities of the human body, including posture maintenance, temperature regulation, organ protection, metabolic function, etc. [301–303]. Through intramuscular injection, mRNA preparations are efficiently delivered to muscle tissue and expressed as expected proteins. This delivery method not only promotes rapid drug absorption and distribution but also enhances immune responses because muscle tissue is rich in antigen-presenting cells. During the COVID-19 pandemic, many world-renowned biotech companies such as Moderna, BioNTech, Pfizer, and CureVac have developed a large number of mRNA vaccines in response to SARS-CoV-2 and its variants [304–306]. Through intramuscular injection, these vaccines deliver the spike protein to stimulate the immune system, leading to the production of specific antibodies. Multiple research studies have confirmed the efficacy, tolerability, and safety of the mRNA vaccines after intramuscular administration. A report from Moderna indicated that mRNA-1273 (dose: 100 μg) had a protection rate of up to 94.1% against SARS-CoV-2 after two doses, with moderate reactogenicity but no significant side effects [307]. Another study from BioNTech claimed that their jointly developed BNT162b2 mRNA vaccines (dose: 30 μg) had an efficacy rate of 95% in preventing SARS-CoV-2, also using intramuscular injection [308]. The success of the two vaccines has drawn worldwide attention to mRNA technology and also promoted research on mRNA delivery systems [309–312]. For example, Zhang et al. created a series of 4N4T-derived LNP delivery systems and observed that MIC1 and MIC2 groups had higher in vivo delivery efficacy than the SM-102 lipid used by Moderna (Fig. 21f) [313]. Importantly, mRNA vaccines prepared using MIC1 or MIC2 produced high antibody titers and Th1-based T cell responses to fight against SARS-CoV-2 variants (Fig. 21g, h). Another study used combinatorial chemistry to identify a new ionizable lipid called iso-A11B5C1, which also showed superior performance in muscle tissue transfection compared to SM-102 [314]. Although many studies have developed ionizable lipids with different chemical structures, mRNA targeting muscle is mainly achieved through intramuscular administration rather than intravenous administration.

mRNA sequences, mRNA carriers, targeting mechanisms, biological barriers, and targeting strategies are interconnected key factors that collectively determine the expression level, site of expression, and therapeutic efficacy of mRNA. The design of the mRNA sequence is crucial, as it directly impacts mRNA stability, translation efficiency, and immunogenicity, all of which influence therapeutic outcomes. During the development of mRNA carriers, optimization should be carried out according to specific targeting mechanisms, including passive targeting, endogenous targeting, and active targeting. Passive targeting relies on natural physiological processes, endogenous targeting achieves precise delivery through specific biological markers or receptors in the body, and active targeting involves functionalizing the carriers by adding specific ligands. However, to achieve effective targeted delivery, multiple biological barriers must be overcome, including the circulatory barrier, endothelial barrier, and endosomal barrier, all of which can limit the efficiency of mRNA delivery. By comprehensively considering these factors, the precision of mRNA therapy is likely to be improved, ensuring that therapeutic mRNA reaches its intended site of action and minimizing off-target effects.

## Conclusion and Future Perspectives

As a cutting-edge therapeutic approach, mRNA technology offers many advantages, including customizability, shorter production cycles, and potentially lower toxicity. These advantages provide strong motivation for developing targeted therapeutic strategies. Considering the importance of targeting strategies, this review offers a comprehensive overview from three primary viewpoints. Firstly, we elucidated the mechanism of mRNA nanomedicines, focusing on the structural modifications of mRNA and the delivery carriers associated with targeted therapies. In the subsequent section, we highlighted the differences between passive targeting, endogenous targeting, and active targeting and also described the biological barriers that mRNA nanomedicines may encounter in vivo. In the third part, we extensively discussed targeting strategies related to mRNA technology, which can enable the precise delivery of mRNA to organs like the heart, liver, spleen, lungs, brain, and others.

Currently, mRNA-related targeting strategies mainly involve altering administration methods, optimizing LNP components, developing novel delivery systems, and modifying ligands. Despite significant achievements, these targeting strategies still face numerous challenges. For instance, altering the administration methods may amplify treatment complexity, requiring the creation of additional drug delivery devices or systems. This could lead to increased costs associated with treatment plans and impose a greater burden on patients. Simultaneously, employing unconventional routes of drug administration may provoke discomfort or aversion in patients, thereby impacting treatment adherence. Secondly, ligand modification on the surface of carriers can affect the stability of the resulting nanomedicine, which involves various factors such as the choice of chemical reactions, interactions between ligands and carriers, solvent selection, and meticulous control of production processes. Thirdly, nanoparticles may interact with various proteins, which can lead to the formation of a more diverse protein corona. Besides, the existing corona isolation techniques have low throughput, long processing times, and require expensive equipment and consumables, which constrains their application and scalability in large-scale production. Finally, although a large number of mRNA-based targeting strategies have been reported, the translation from laboratory to clinical settings is limited, primarily due to intracellular escape failures, potential immune reactions, and off-target toxicity.

To achieve more precise mRNA delivery, we propose the following opinions on future research. (1) For specific diseases, it is necessary to consider the integration of mRNA nanomedicines with more precise delivery platforms, such as microneedles, capsules, gels, and microfiber systems [315–319]. As a typical example, one study delivered mRNA-loaded exosomes into the dermis using hyaluronic acid microneedle patches, inducing collagen synthesis and significantly improving skin aging [320]. (2) Explore more on the targeting mechanism of mRNA. Although there are many reports related to targeted delivery at present, explanations of the mechanism are rare, and further improvement is needed in related work. (3) The targeting strategies for mRNA should progressively shift from the organ level to the cellular level. Generally, most targeting strategies are aimed at entire organs or tissues, which can lead to the widespread distribution of drugs in the body and increase the risk of adverse reactions. Many studies have demonstrated selective organ targeting through the conjugation of ligands or antibodies to PEGylated lipids. We believe that conjugating multiple ligands or antibodies to PEGylated lipids is a promising targeting strategy, with the potential to achieve cell-level targeting. Future research should focus more on targeted delivery to specific cells, which can reduce the non-specific distribution of mRNA nanomedicines in the body and maximize therapeutic efficacy. (4) The targeted delivery strategies for mRNA nanomedicines need to be tested in different disease models and higher-order biological species. Due to disease variability, the composition or types of protein coronas in vivo can vary significantly, making it essential to consider different disease models when investigating protein coronas. Additionally, variations in blood flow and protein composition between different species mean that data from animal models may not directly translate to humans. Some studies have confirmed that targeted delivery results demonstrated in rodents or other small organisms may not be replicable in more advanced species. (5) Investigating protein corona information may bring greater advantages to targeted therapy. It has been demonstrated that utilizing protein coronas can be used for disease diagnosis and reduce off-target effects. Through a deeper understanding of the composition and characteristics of protein corona, it is helpful in optimizing targeted delivery systems, enhancing the affinity between drugs and target cells, and improving the expected outcomes.

In special mRNA therapies, some tools do not directly participate in mRNA expression but play a significant role in mRNA regulation, such as the Aptamer–DNAzyme system [321, 322]. Aptamers, as highly specific molecular probes, can target particular cells or molecules, enabling precise delivery and regulation. DNAzymes, on the other hand, are nucleic acid molecules with enzymatic activity that can catalytically cleave-specific RNA sequences or regulate gene expression. By combining aptamers with DNAzymes, highly specific regulatory tools can be constructed to achieve precise control over mRNA regulation.

Although tools like regulatory and delivery systems are essential for mRNA regulation and expression, their side effects should not be overlooked, especially the risks associated with delivery systems. Common adverse reactions include local inflammation, allergic responses, and potential off-target effects, all of which may lead to discomfort. Reports indicate that some lipid-based carriers can induce toxicity due to their cationic or PEG components [323]. To address these concerns and achieve more precise targeting at the organ or cellular level, researchers are exploring advanced delivery strategies, such as ligand-mediated targeting and stimuli-responsive carriers. Furthermore, fine-tuning the physicochemical properties of delivery systems can also improve their biocompatibility and targeting efficiency, paving the way for safer and more effective therapeutic outcomes.

Another important point is that one of the most promising directions for mRNA nanomedicines is the development of personalized treatment strategies tailored to individual patients. With advancements in genomics, transcriptomics, and proteomics, patients can gain a comprehensive understanding of their genetic characteristics, disease stages, and physiological conditions, enabling the identification of key proteins that are deficient or functionally impaired. By integrating artificial intelligence and machine learning into mRNA drug development, the design of mRNA sequences can be optimized, and the expression efficiency and stability of therapeutic proteins can be significantly improved. Such innovations can not only address the diverse needs of patients, but also drive the advancement of precision medicine.
